# Self‐Adaptive Reflectance Film for Passive Temperature Regulation in Diverse Environments

**DOI:** 10.1002/advs.202415121

**Published:** 2025-04-26

**Authors:** Shuo Yang, Sizhe Tang, Yufeng Cai, Zhiming Ye, Xiangbin Zou, Xueyu Yuan, Yujie Song, Bing Li, Dongyan Tang, Ming Liu

**Affiliations:** ^1^ School of Chemistry and Chemical Engineering Harbin Institute of Technology Harbin 150001 P. R. China; ^2^ State Key Laboratory of Special Functional Waterproof Materials Beijing Oriental Yuhong Waterproof Technology Co. Ltd Beijing 101111 P. R. China; ^3^ Engineering Laboratory of Advanced Energy Materials Ningbo Institute of Materials Technology & Engineering Chinese Academy of Sciences Ningbo 315201 P. R. China; ^4^ Advanced Energy Science and Technology Guangdong Laboratory Huizhou 516000 China; ^5^ State Key Laboratory of Advanced Welding and Joining Harbin Institute of Technology Harbin 150001 P. R. China

**Keywords:** dynamic reflectance tuning, janus porous film, passive temperature regulation, radiative cooling

## Abstract

Passive thermal‐regulation strategies have become increasingly important due to the strain alleviated on power grids for temperature management. Designing a system capable of automatically switching between cooling and heating modes in response to changing ambient conditions presents several specific challenges that engineers and researchers are actively addressing. In this research, a CaCl_2_ incorporated PNIPAM coated fluorinated poly(aryl ether) (FPAE) porous film with tunable reflectance is developed. The aim is to mitigate the reliance on active cooling systems, which consume significant amounts of energy. The moisture‐temperature dual sensitive film exhibits a tunable reflectance range between 91.1% and 39.1% via phase change of the PNIPAM layer. Coupled with an infrared emissivity of 96.0%, a daytime cooling of 10 °C compared to the control experiment is achieved. Coating the film with a photothermal layer results in an adaptive Janus film that is capable of autonomous switching between heating and cooling, and demonstrates a heating of 22.5 °C in a cold environment. The facile preparation method, excellent cyclic stability, mechanical properties, and UL‐94 V‐0 rating enable promising applications of the smart film under diverse living environments.

## Graphical abstract

The rising demand for space cooling calls for innovative passive thermal systems. Traditional radiative cooling materials lack efficiency and alignment with cooling needs, limiting their energy‐saving. A smart film (PFSF) that integrates radiative cooling, reflectance tuning, and phase transition, offering adaptable temperature control across climates is introduced. This is a key advance in passive thermal management with strong practical potential.

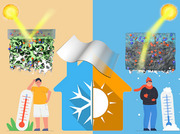

## 1 Introduction

A large proportion of daily energy consumption is used for space cooling and heating to achieve thermal comfort.^[^ ^1^ ^]^ To tackle the escalating energy demand, passive thermal management strategies are being explored extensively as promising alternatives to ensure greater comfort without the parallel growth in energy consumption and related emissions.^[^ ^2^ ^]^ Radiative cooling is seen as an appealing passive cooling approach by radiating heat into the cold sink of outer space.^[^ ^3^ ^]^ In the realm of radiative‐cooling materials, multilayer photonic structures,^[^ ^4^ ^]^ nanocomposite film,^[^ ^5^ ^]^ metamaterials,^[^ ^6^ ^]^ and porous polymer structures^[^ ^7^ ^]^ are commonly adopted. Traditional radiation‐cooled materials usually have high reflectance in the visible and near‐infrared region of the electromagnetic spectrum,^[^ ^8^ ^]^ and demonstrate strong emissivity in the middle and far infrared.^[^ ^9^ ^]^ These performance characteristics ensured effective cooling in the hot daytime, but became a disadvantage in a colder environment where heat conservation is preferred.^[^ ^10^ ^]^

Unbiased cooling or the poor synchronization between cooling demand and cooling ability limits the energy‐saving potential and the application capacity of a typical radiative cooling material.^[^ ^11^ ^]^ For instance, cooling demand is mainly concentrated in the daytime,^[^ ^12^ ^]^ however, the cooling effect is hampered by the strong solar radiation.^[^ ^13^ ^]^ In contrast, radiative cooling materials demonstrate a greater cooling effect at night when cooling needs are not the priority.^[^ ^14^ ^]^ Moreover, passive cooling materials that rely on high reflectance to manage heat become less effective^[^ ^15^ ^]^ or even obsolete in cold weather when low reflectance is preferred to maintain warmth.^[^ ^16^ ^]^

Recent research works shift toward dynamic regulation of temperature management systems that involve the real‐time adjustment of cooling/heating mechanisms based on immediate needs or environmental conditions.^[^ ^17^ ^]^ Janus films composed of mechanically bonded photothermal and radiative layers were fabricated to integrate cooling and heating.^[^ ^18^ ^]^ However, the manual switching required by such Janus structures poses difficulties for large‐scale applications.^[^ ^19^ ^]^ Mandal et al. reported a porous polymer exhibiting large reversible changes in optical reflectance for seasonal or diurnal temperature management.^[^ ^20^ ^]^ Nonetheless, the working range and capacity are fundamentally limited by the complex leak‐proof liquid circulation system. Li et al. developed a polyurethane nanofiber film with a reversible reflectance regulation range of 61.1% by manual or electrodynamic stretching.^[^ ^21^ ^]^ Temperature‐controlled materials with self‐regulating capabilities and zero energy input were also being explored.^[^ ^22^ ^]^ Wu et al. designed an adaptive sandwich film using thermochromic PNIPAM hydrogel and PVDF film.^[^ ^23^ ^]^ The properly sealed film achieved 1.8 °C for cooling and 4.3 °C for heating.^[^ ^24^ ^]^

In nature, the evolution of plants to thrive in their specific habitats has inspired numerous application designs. This work took inspiration from the skeleton flower (*Diphylleia grayi*) that exhibits moisture adaptation to enhance its survival. The air pockets within the petals reflect light giving the flower a white appearance when dry. When it rains, the air pockets are filled with water, and the similar refractive index between water and the cytolymph (watery cell‐sap) allows light to pass through, and change the appearance to transparent.^[^ ^25^ ^]^ An adaptive polymer film that switches from white to translucent upon moisture absorption is fabricated using fluorinated poly(aryl ether) (FPAE). A layer of PNIPAM precipitated on a porous FPAE film through a non‐solvent exchange process. Calcium chloride (CaCl_2_), a hygroscopic salt that can take up water at relative humidities beyond 35% was then deposited evenly on the film to act as a water source to swell the PNIPAM hydrogel layer.

The proposed strategy synergistically integrates reflectance tuning, radiative cooling, and phase transition interaction, to form a cohesive protocol design to achieve on‐demand. The hygroscopic CaCl_2_ bypassed the need for encapsulation for PNIPAM. An optical transmittance ranging between 91.1% and 39.1% through absorption of moisture from the ambient according to temperature fluctuation was obtained based on a mechanism similar to the skeleton flower. Outdoor tests conducted in locations of distinct latitudes demonstrated an effective cooling capacity, up to 10 °C cooling was achieved on a hot summer day. Coating a photothermal layer on one side of the film endowed the film with excellent heating ability on a cold day without human intervention. The save in energy consumption through harnessing the multiple cooling mechanisms exhibited by the film in 9 cities with distinct weather conditions around the world estimated by EnergyPlus demonstrates the versatility of the film.

## 2 Results and Discussion

### 2.1 Preparation and Characterization of PFSF Porous Film

**Figure 1:**
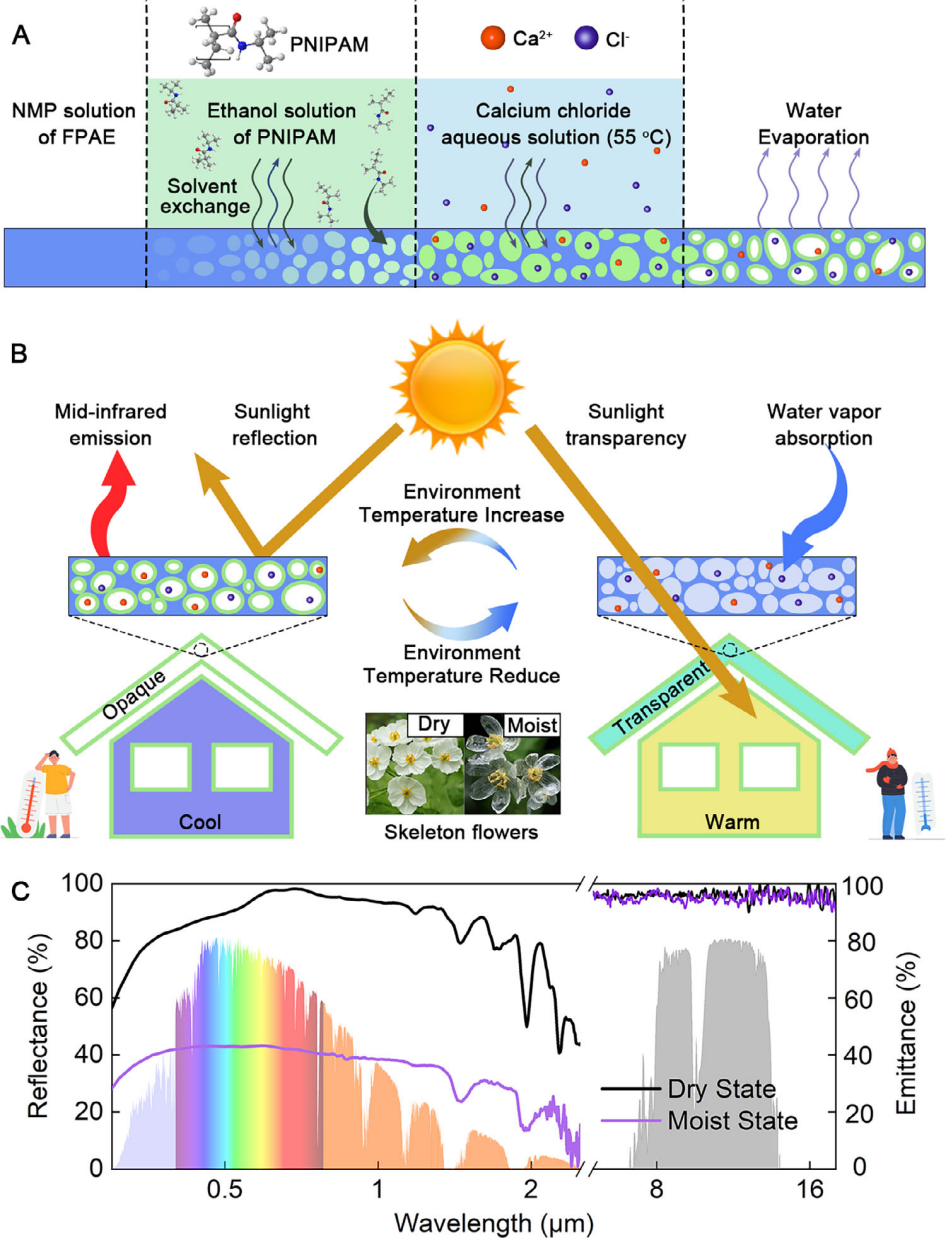
The schematic illustration for the fabrication and the SEM images of PFSF films. A) Schematic illustration for the preparation process of the PFSF film via non‐solvent induced phase inversion method. B) Schematic of the different states of the PFSF film under high environmental temperature (left) and low environmental temperature (right). C) Reflectance (0.3–2.5 µm) and emittance (6–18 µm) spectra of the PFSF film. The solar spectrum (colored shaded area), and atmospheric transmittance window (grey shaded area) are plotted for reference.

**Figure** **1**A illustrates the porous film preparation process. FPAE was dissolved in NMP and cast onto a glass substrate. The FPAE‐coated glass substrate was then subjected to the non‐solvent‐induced phase inversion process by soaking in an ethanol solution containing PNIPAM for 10 min. The film was then immersed in an aqueous solution containing 20 wt.% CaCl_2_ at 55 °C for 1 h to introduce hygroscopic properties by the nanoscale dispersion of salt ions. A self‐standing CaCl_2_ containing PNIPAM‐coated porous composite film (PFSF film) was obtained upon drying.

Figure 1B provides a schematic for the temperature and moisture‐triggered transmittance change of PFSF film. The dry PFSF film has an opaque white appearance and a reflectance of more than 91%, providing effective cooling in the visible to near‐infrared light (NIR) range (Figure 1C). When being exposed to lower temperatures, the hygroscopic of CaCl_2_ with high water affinity absorbs moisture rapidly from the surrounding air to swell the PNIPAM which in turn fills up the air pockets. The film turns translucent along with the reduction in the refractive index mismatch. The average reflectance dropped to 39%, allowing solar light to pass through the film providing radiant heating to the enclosure/object beneath.

### 2.2 Material Design and Characterization

**Figure 2:**
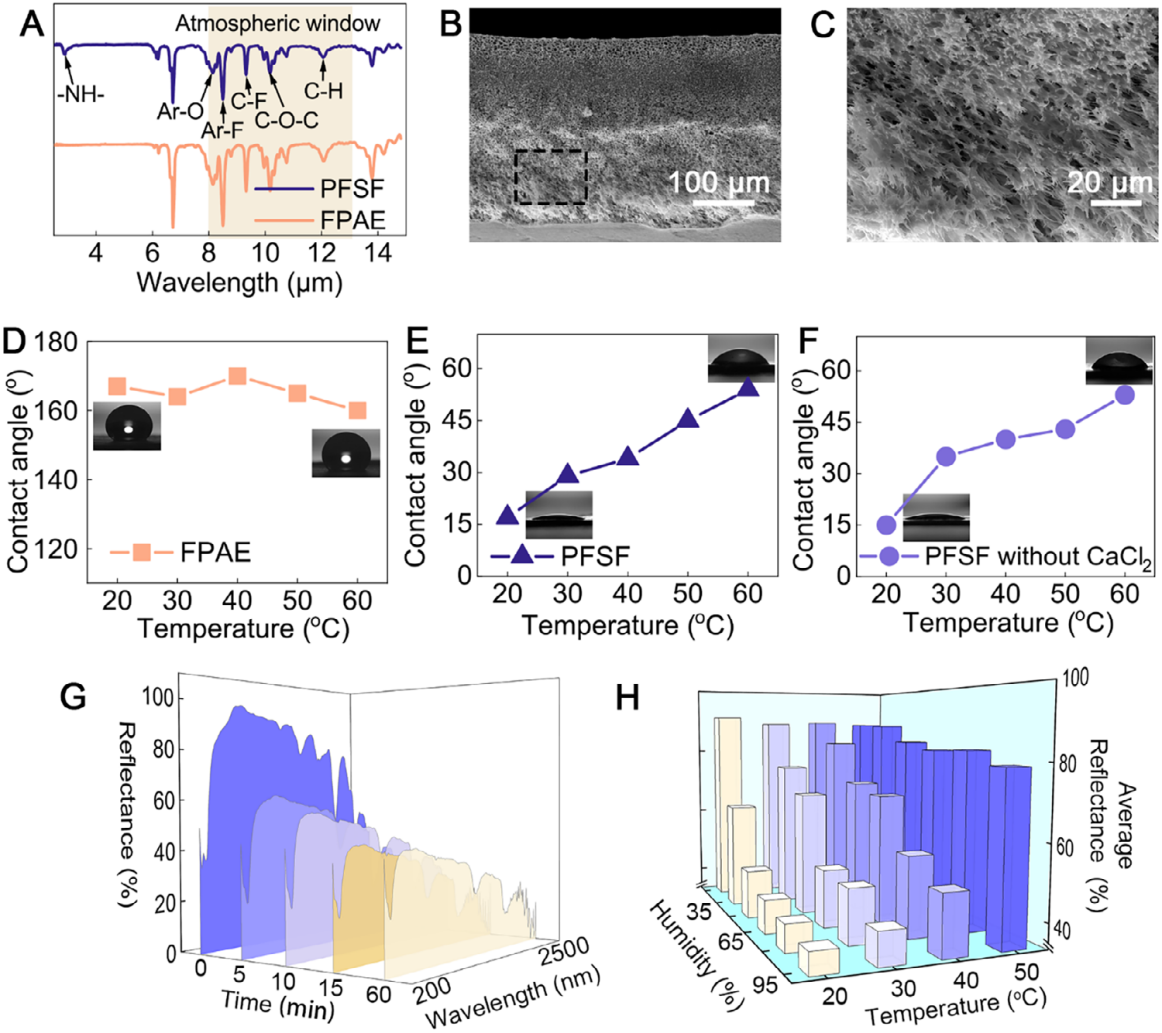
Characterization of PFSF film. A) FTIR‐ATR spectra of PFSF and FPAE. The SEM images of B) the cross‐section of the PFSF film and C) the enlarged image. The water contact angle of D) FPAE, E) PFSF, and F) PFSF (without CaCl_2_) film at different temperatures. G) The reflectance spectra as a function of time for PFSF film stored at 20 °C and 95% RH. H) The average reflectance of the PFSF film after storage at 20 °C and 95% RH for 60 min.

Molecular vibration theory and Mie scattering theory serve as fundamental principles for developing passive cooling materials and systems that effectively regulate heat transfer through radiation and reflection, enhancing thermal comfort and energy efficiency across various applications. The molecular structure and microstructure of the PFSF films were designed according to the theories aforementioned to achieve efficient cooling via mid‐infrared emission and visible‐near‐infrared reflection, respectively. In addition, the strong spectral response at the atmospheric window (8–13 µm) will endow an organic material with emission properties that favor cooling. The ATR‐FTIR spectra (**Figure** **2**A) show clearly that the stretching vibrations of C─F, C─H, and C─O─C bonds in the 8–13 µm band are contributed by the FPAE skeleton. Due to the strong coupling of C─F, C─H, and C─O─C, the average emittance of PFSF film reaches 96.1% (dry state) and 94.9% (moist state) in the atmospheric window (Figure 1C). In addition, FPAE is chosen as the skeleton for its excellent chemical and physical stability.

The porous microstructure of a film enhances its high reflectance in the NIR spectrum by providing multiple scattering sites that effectively reflect NIR light and improve thermal management capabilities. The porosity of the PFSF film was obtained by adjusting the conditions employed for non‐solvent‐induced phase conversion (Figure 2B). The pore size is controlled between 1 and 3 µm (Figure 2C) because pore size exceeding the size of the swelled PNIPAM particle will leave hollow space within the film and reduce the reflectance. The introduction of the PNIPAM layer turned the intrinsically superhydrophobic FPAE film to hydrophilic (Figure 2D), favoring water absorption. Interestingly, the hydrophilicity of the film dropped when being tested from 20 to 60 °C. This feature prevents rapid moisture absorption by CaCl_2_ at higher temperatures when an opaque film is preferred for cooling (Figure 2E,F). Below the Lower Critical Solution Temperature (LCST), the amide groups (─CONH) in PNIPAM form hydrogen bonds with water, providing the hydrogel with water compatibility. The hydrogel effectively stores water and enables the PFSF film to transition from the cooling state to the heating state. Above the LCST, the hydrogen bonds between the amide groups and water molecules are disrupted, and the hydrophobicity of the isopropyl side chains becomes dominant. The hydrogel resists water absorption, allowing the PFSF film to perform radiative cooling.

### 2.3 Reflectance and Hydrophilicity Regulation of PFSF Films

A thin layer of PNIPAM was introduced as a reflectance control unit in the PFSF film. To determine the reflectance tuning conditions and the speed of reflectance change, orthogonal tests were carried out taking temperature and humidity as variables. The reflectance was recorded at 0, 5‐, 10‐, 15‐ and 60‐min intervals at temperatures of 20, 30, 40, and 50 °C, and relative humidity (RH) of 95%, 80%, 65%, 50%, and 35%. The reflectance obtained for the PFSF films stored at 20 °C and 95% RH at a different time interval (Figure 2G) shows that the average reflectance decreased drastically from 91.1% to 57.1% during the initial 5 min, and plateaued at 39.1% after 30 min. The results under all testing conditions are presented in Figures S6–S9 (Supporting Information).

The PFSF film can be tuned dually by humidity and temperature by leveraging the thermoresponsive behavior and optical properties of PNIPAM demonstrated under dry and wet states. As shown in Figure 2H, humidity demonstrates a dominant influence on the average reflectance at 20 °C with reflectance recorded as 63.4% and 39.1% under 35% RH and 95% RH, respectively. The reflectance drops concurrently as the CaCl_2_‐loaded PNIPAM gradually turns translucent upon moisture absorption from the surroundings. By keeping the RH constant, increasing the temperature generally leads to higher reflectance due to moisture evaporation from the PNIPAM layer, leaving an opaque film. At 50 °C, a temperature well above the LCST of PNIPAM, the influence of humidity becomes insignificant when PNIPAM becomes hydrophobic.

### 2.4 PFSF Film for Passive Cooling

The autonomous reflectance changes according to the surroundings of the PFSF films make it an ideal temperature regulation material for buildings, vehicles, and other objects. During daytime, the PFSF films achieve passive indoor space cooling via two ways, reflection of solar radiation and the endothermic evaporation of water from the PNIPAM layer as illustrated in Figure S19 (Supporting Information). When temperature drops or during nighttime, moisture uptake of the hygroscopic CaCl_2_ happens and the film replenishes water reserve for evaporation and heat dissipation when the heat needed for desorption is conveniently provided by solar radiation. At temperatures below 20 °C, the translucent film due to swelled PNIPAM allows sunlight radiation to pass through hence reducing the energy consumption needed for heating. The average reflectance of the PFSF film is dependent on the level of humidity at temperatures between 20 and 40 °C. The film has a transparent appearance or lower reflectance when the humidity is high, such as on rainy or foggy days. When the humidity is low, such as on hot sunny days, the film turns opaque and the high reflectance provides a radiation‐cooling effect for the object underneath it. Further increasing the temperature to above 40 °C, the high reflectance dehydrated and superhydrophobic white film provide continuous radiation cooling despite humidity fluctuation. It is worth noting that the reflectance changes are spontaneous behaviors of the PFSF films that do not require additional equipment or input energy to control. The PFSF film demonstrates better performance and lighter weight than commonly used PNIPAM hydrogel (Figures S12–S14, Supporting Information).

### 2.5 Field Test for Temperature Regulation Evaluation of PFSF

**Figure 3:**
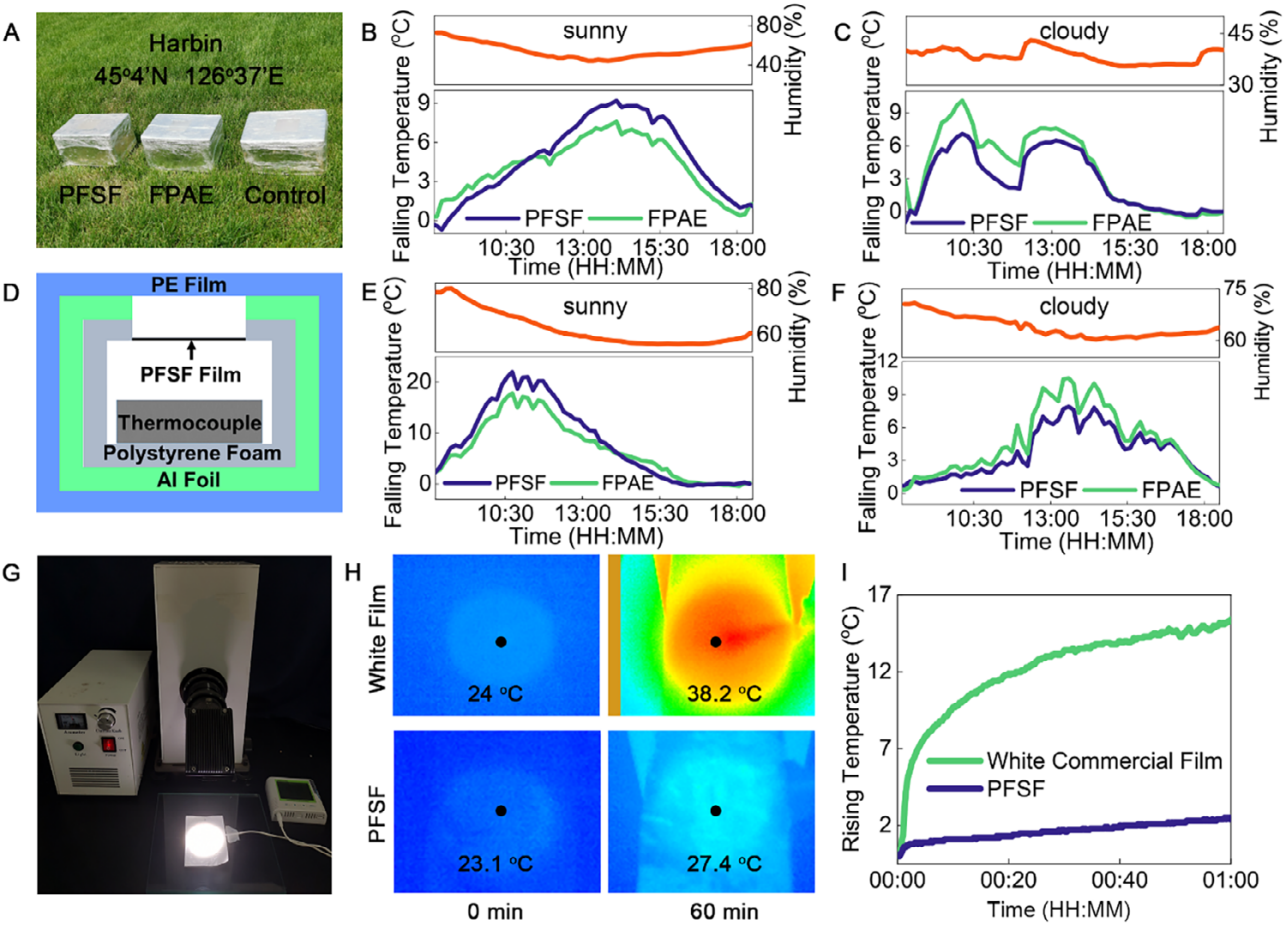
Field test performance of PFSF film. (A) Digital image of the cooling test setup placed on an open square in Harbin. Temperature drop recorded on (B) sunny day, (C) cloudy day, in Harbin city, China. (D) Schematic of the cooling test setup. The Temperature drop recoded on (E) sunny day, (F) cloudy day, in Fuzhou city, China. (G) The in‐house test simulates solar radiation using a high‐power xenon lamp. (H) Thermal infrared images of PFSF film and commercial white film after illumination. (I) Rising temperature curve of the PFSF film and commercial white film during the illumination.

Outdoor experiments are conducted using an in‐house build setup to evaluate the system performance during real‐life scenarios. As shown in **Figure** **3**D, the setup consists of a polyethylene foam box as the main body, aluminum foil was affixed to the exterior walls to reflect solar heat radiation, and the surface was wrapped by a PE film to prevent heat convection. A 10 cm × 10 cm window was cut out from the lid and replaced by the test sample. The temperature change was recorded by thermocouples placed inside the box. The tests were conducted on 26 July and 21 September 2023 in Harbin city (45°4′N, 126°37′E) (Figure 3A), northern China and 15 August and 19 September 2023 in Fuzhou city in southern China (26°4N’,119°20E’) (Figure S15, Supporting Information).

The average interior temperatures of the setups were recorded in both Harbin and Fuzhou from 9:00 to 18:00. On a hot sunny summer day in Harbin, PFSF shows better cooling effect than FAPE. The films with high reflectance effectively reduced the interior temperature of the FPAE and PFSF covered set up by 5.2 °C and 6.0 °C below that of the control setup, respectively, as shown by the representative data in Figure 3B. The test conducted in Fuzhou showed similar results, the recorded average temperatures were 8.5 °C and 10.0 °C lowered than that of the control set up, respectively Figure 3E. The incorporation of hypogenic components endows PFSF multiple cooling mechanisms including radiative cooling, and water evaporation (The enthalpy of evaporation of PFSF film is 3.5 KJ/g, Equation S3, Supporting Information) with minimal active energy input over a relative humidity range. On days with lower ambient temperature or during cloudy days, PFSF film exhibits different cooling capacity in both cities. As shown Figure 3C,F, from 9:00 to 18:00, FPAE is more superior in terms of cooling ability. The interior temperature of PFSF covered set up was 1.3 °C and 1.4 °C higher than the one covered by FPAE in Harbin and Fuzhou, respectively. The reduced cooling ability of PFSF at lower temperature or on cloudy days was caused by the incomplete evaporation of the moisture inside the PNIPAM hydrogel which kept the PFSF in the translucent state allowing solar radiation to partially pass through and heat the enclosure beneath.

Laboratory tests were used to compare the cooling performance of the PFSF film with commercial white polymer films. As shown in Figure 3G, a xenon lamp was used to simulate the solar radiation and cast a uniform light irradiation on test surfaces. The temperature measuring probe is placed at the bottom of the film to monitor the temperature change. From the infrared temperature photos Figure 3H, it can be seen that after 60 min of irradiation, the surface temperature of the white film increased by 15.2 °C, while the surface temperature of the PFSF film only increased by 2.4 °C (Figure 3I). This is because the vast majority of light radiation was reflected by the large number of internal porous within the PFSF film, while the commercial white film can only reflect the visible region of the radiation.

### 2.6 Energy Consumption Simulation

**Figure 4:**
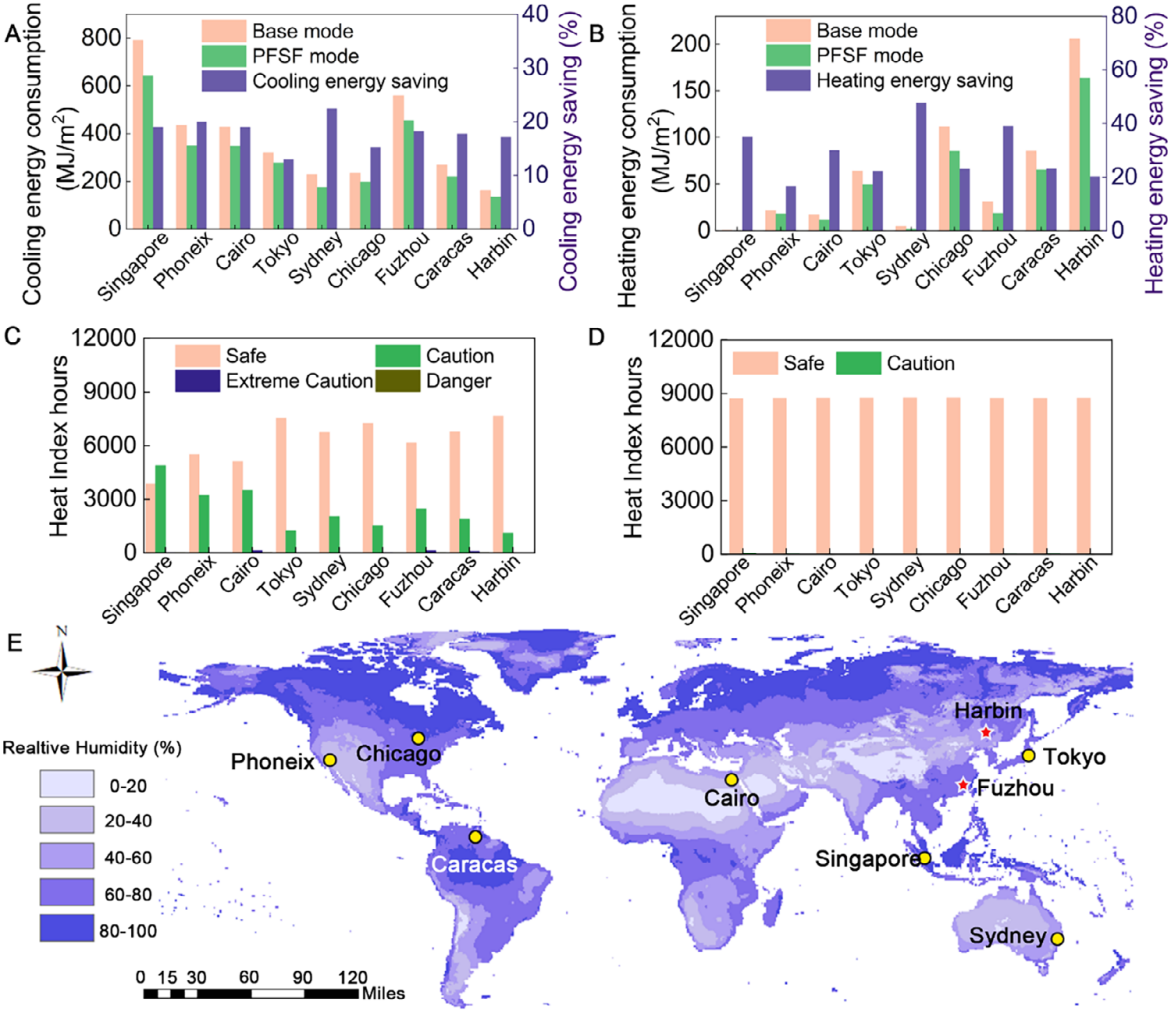
Simulation for energy‐saving performance in buildings. Simulation of energy consumption and energy saving for a building covered by PFSF during a period of one year (A) for cooling, (B) for heating. Heat index hours of a building (C) without PFSF film, (D) cover with PFSF film. (E) Geographic distribution of world average annual relative humidity and the cities shortlisted for simulation.

To evaluate the energy‐saving potential of PFSF films, the film was applied as the roof surface of a typical building model, and the energy consumption was calculated (Figure S18, Supporting Information). Energyplus was used to simulate the energy consumption for temperature regulation of a PFSF covered building in 9 cities from different climate zones around the world (**Figure** **4**E). The simulation results show that the cooling energy consumption was significantly reduced in all the cities shortlisted. Cooling energy consumption of the PFSF film covered buildings was reduced by 13% to 22.4% compared to the non‐covered buildings (Figure 4A). The results also show that PFSF films can effectively reduce energy needed for heating. For cities such as Harbin, where the outdoor temperature can be as low as ‐30 °C, heating energy consumption can be reduced by 20.3%. In regions with warmer winters, such as Singapore and Cairo, heating energy consumption can be reduced by 35% to 47.9% (Figure 4B).

The heat index, or apparent temperature, represents how the human body perceives temperature by combining air temperature and relative humidity. This interaction is crucial for understanding human comfort in various environments, and is categorized into three ranges: caution (26.7 °C to 32.2 °C), extreme caution (32.2 °C to 39.4 °C), and danger range (39.4 °C to 51.7 °C) (Figure 4C). According to the simulation results, over the course of a year (8760 h), the average interior temperature of the building maintains in the safe range (≤ 26.7 °C) for roughly 5100 to 7500 h. In cities such as Singapore and Phoenix, only 3842 and 5511 h, respectively. When the PFSF film is applied as the roof surface of the building, the time spent in the safe range for all cities increase to ≈8700 to 8760 h, credited to the passive cooling effect. The time spent in extreme caution or dangerous range is eliminated (Figure 4D). The results demonstrate that adaptive temperature regulation for buildings based on immediate needs or environmental conditions is achievable.

### 2.7 Physical Properties of PFSF Film

**Figure 5:**
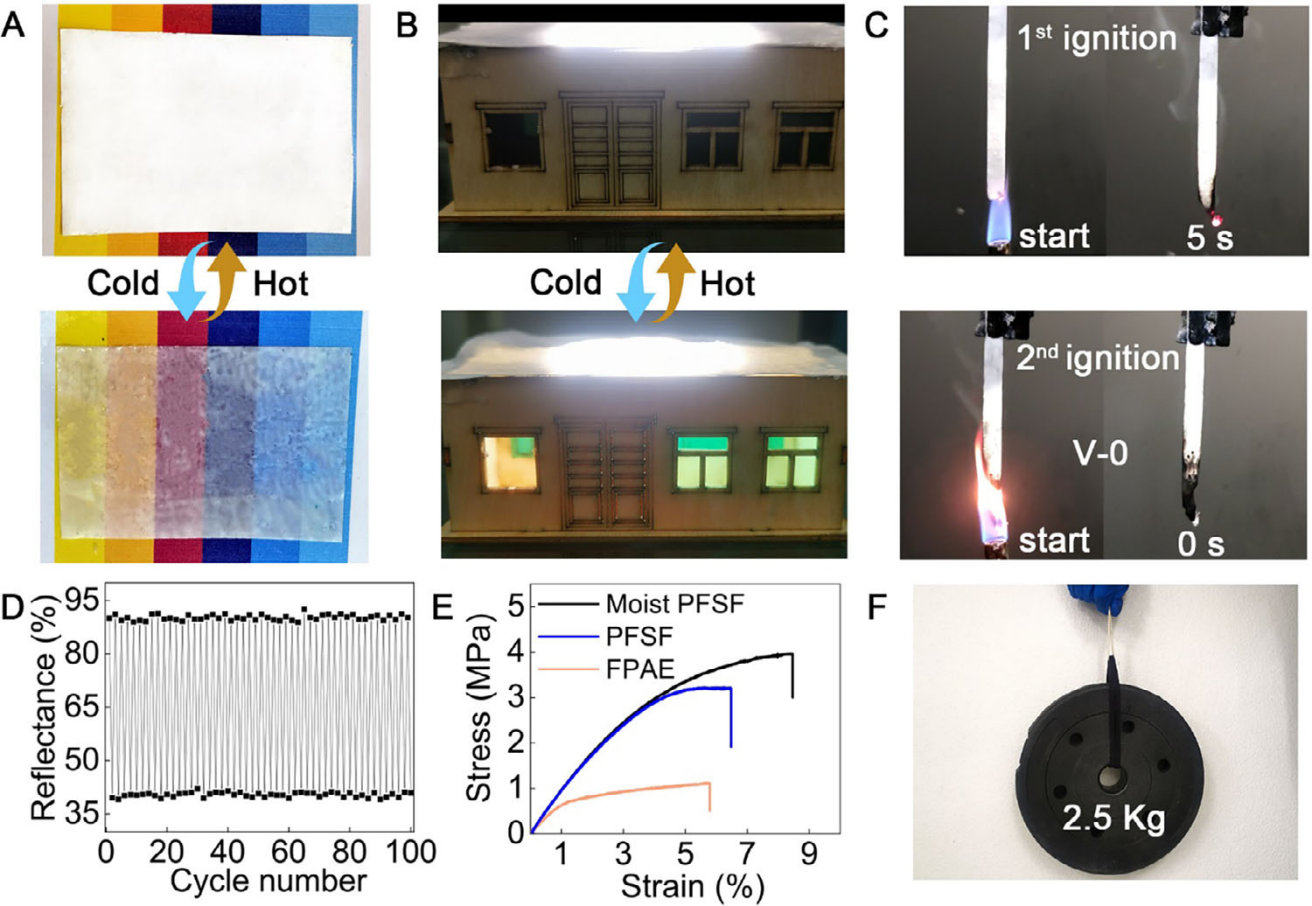
The physical appearance and properties of PFSF film. A) PFSF film transforms from opaque to translucent upon moisture absorption from the surroundings in 20 min (20 °C, RH: 95%). B) Digital photo of the model house cover by dry state PFSF and moist state PFSF film. C) Process of flame‐retardant testing of PFSF films. D) The average reflectance of PFSF films cycling between dry and moist states. E) Mechanical strength of FPAE, PFSF, and moist PFSF film. F) Digital photo of PFSF film (thickness: 0.848 mm) pulling up a 2.5 Kg dumbbell plate.

In addition to temperature regulation, PFSF film in the wet state can also be utilized to reduce energy consumption for lighting. The CaCl_2_ rich PNIPAM absorbs moisture at night and increases the transparency of the film allowing partial passing through of the external light sources, such as moonlight or street light. As demonstrated in **Figure** **5**A, the PFSF film changes from opaque to translucent revealing the image placed underneath clearly after placing at 20 °C and 95% RH for 30 min. The interior of the model house was light up by external light source when the dry PFSF film absorbed moisture and became translucent (Figure 5B).

Figure 5C shows the physical appearance of PFSF film after two ignitions by a blowtorch according to the UL‐94 flame retardant test. After ignition, the flame was extinguished within 10 s without melting and dripping, and the results met the optimal fire rating V‐0.5 groups of parallel samples were tested, and the test results were shown in Table S1 (Supporting Information).

The PFSF film was hydrated at 20 °C, 95% RH for 30 min and dehydrated in a preheated oven at 60 °C for 30 min repeatedly to investigate the cycling stability. The reflectance dropped and revert recorded showed the reflectance adjustment range was maintained at ≈52% after 100 moist‐drying cycles, indicating a favorable cyclic stability of the film (Figure 5D).

The mechanical properties of PFSF films were characterized by a universal testing machine. The fracture tensile strength of FPAE, and PFSF in the dry and wet states are determined as 1.11, 3.21, and 4.01 MPa (Figure 5E), respectively. The PNIPAM coating reinforced the porous structure of FAPE and enhanced the tensile strength. The tensile strength was further improved when the PFSF film absorbed moisture from the surroundings and formed hydrogen bonds within the network. The swelled PNIPAM hydrogel filled the empty pores also leads to better flexibility as reflected by the increase in the stress‐strain behavior. The flexibility and processability of PFSF film were demonstrated by bending and shaping. (Figure S20, Supporting Information). A piece of PFSF film with a thickness width of 0.858 mm can pull up a 2.5 Kg dumbbell plate (Figure 5F), showing excellent mechanical strength.

### 2.8 PFSF‐B Film for Cooling/Heating Dual Regulation

**Figure 6:**
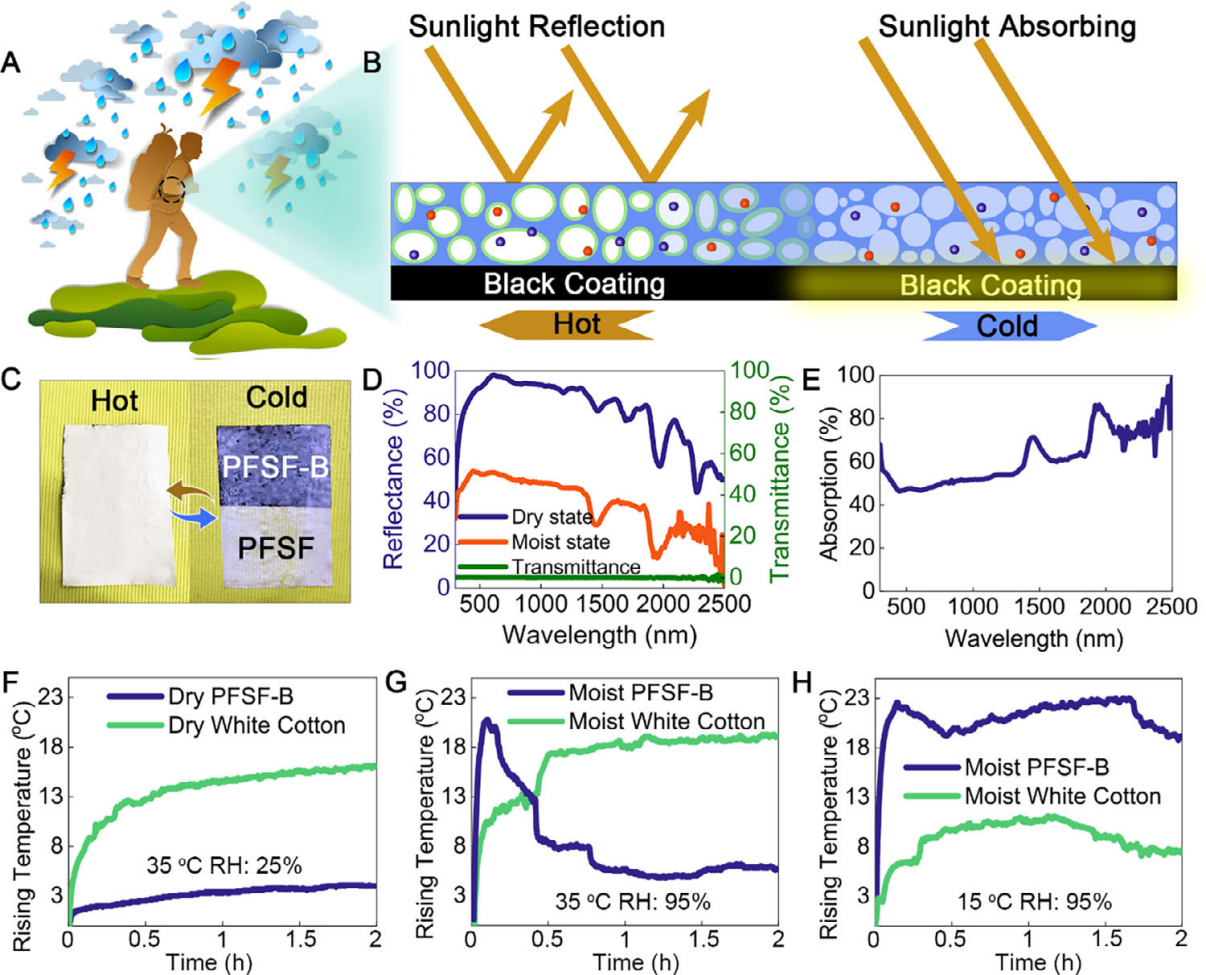
Mechanism and performance of dual function PFSF‐B film. A) Extreme sports such as mountaineering will encounter extreme weather such as short‐term heavy precipitation. B) A schematic diagram of a PFSF‐B film reflects solar radiation under the dry state and, absorbing solar radiation under the moist state. C) Digital photos of PFSF film (right) and PFSF‐B film (left) stored under 20 °C, RH: 95% for 20 min. D) Reflectance and transmittance curves of PFSF‐B film. E) Absorption curve of PFSF film. The rise in surface temperature for PFSF film and white cotton subjected to simulated solar light under 35 °C, 25% RH F), 35 °C, 95% RH G), 15 °C, 95% RH (H).

Weather fluctuations during outdoor activities such as hiking pose dangers ranging from discomfort to serious health risks (**Figure** **6**A). An adaptive fabric that can respond dynamically to changing environmental conditions, providing comfort and protection can be an effective solution. Besides reflecting most of the radiation when the solar radiation is strong, absorbing solar radiation in a cold environment is equally essential. A photothermal layer was incorporated beneath the film to harness solar energy, which has been demonstrated as a viable and sustainable solution for light‐to‐heat conversion. The moist translucent film allows partial passing through of the light and is absorbed by the photothermal layer which in turn serves as a heating source. The bilayer film (PFSF‐B) functions similarly to the PFSF film in the dry state and switches to a heat source for the object underneath (Figure 6B and Figures S21 and S22, Supporting Information) upon moisture absorption. As shown in Figure 6C, a piece of dry PFSF‐B placed at 20 °C and 95% RH gradually turned translucent and revealed the black coating at the bottom of the film after 20 min, along with an obvious reflectance drop (Figure 6D). According to the formula: absorption rate = 1 – reflectance – transmittance, the average absorption rate of PFSF‐B was calculated as 51.2% (Figure 6E). A summary of various dynamic radiative cooling materials, the stimuli factors, categories, and applications can be found in Supplementary Table S3.

The surface temperature for PFSF‐B film and white cotton cloth exposed to high‐power xenon lamp radiation was compared. After 2 h of irradiation, the temperature of the PFSF‐B film was 10.6 °C lower than that of the cotton fabric (Figure 6F). The humidity was increased to 95% to simulate (Figure 6G) the situation of short‐term rainfall. The surface temperature of the PFSF‐B increased sharply by 20.7 °C, reaching 55.7 °C within 10 min of exposure. On the other hand, the cotton surface temperature rose slowly with exposure time, and an increment of 17.3 °C was measured after 30 min. In addition, considering that the temperature may drop sharply after rain, PFSF‐B film in the moist state can effectively use the absorbent layer for radiation heating when the environmental temperature drops below 15 °C (Figure 6H). The temperature of PFSF‐B film can be increased by 22.5 °C, reaching up to 37.5 °C in 10 min. With temperatures maintained at 35–38 °C during the 2 h test, PFSF‐B film provided the body with the heat needed under extreme conditions. In contrast, cotton fabrics were heated up at a lower speed and the maximum temperature rise was 10.5 °C. In complex weather conditions for outdoor activities, the film can intelligently switch between heating/cooling to maintain the temperature within a comfortable range, and such materials are expected to be widely used in the field of zero‐energy human temperature control.

## 3 Conclusion

A novel approach was adopted in the fabrication of a mechanically robust PNIPAM hydrogel coated fluorinated polyaryl ether (FPAE) porous film, designed for passive temperature regulation purposes. By integrating hygroscopic CaCl_2_ into the porous network as a moisture absorbent, the phase transition of the PNIPAM hydrogel layer was enabled as an alternative to encapsulation. A large reversible NIR reflectance tuning window (39%–91%) was achieved through the exchange of moisture with the surrounding air, demonstrating an effective approach toward passive cooling. A thermal conversion layer was spray coated on the existing film to obtain a bilayer film composed of a humidity‐sensitive radiative cooling FAPE/PNIPAM top layer and a thermal absorbing bottom layer. The temperature/moisture sensitive free‐standing FAPE/PNIPAM film subjected to outdoor tests exhibited autonomous switching between heating/cooling. An average sub‐ambient temperature drop of ≈10 °C under direct sunlight and a heating of 22.5 °C on a cold day were obtained. The dual‐functional energy reduction through solar energy harvesting and radiative cooling estimated by EnergyPlus has demonstrated the potential of deploying the composite film for buildings, vehicles, and greenhouses in areas with large temperature/moisture fluctuations.

## 4 Experimental Section

### Materials

4,4′‐(hexafluoroisopropylidene)diphenol, decafluorobiphenyl, methyl alcohol, ethanol, N‐methyl‐2‐pyrrolidone (NMP), dichloromethane (DCM), N‐isopropylacrylamide (NIPAM), 2,2′‐Azobis(2‐methylpropionitrile) (AIBN), N,N‐methylenebisacrylamide Bis‐acrylamide (BIS), potassium persulfate (KPS), N,N,N″,N″‐Tetramethylethylenediamine (TEMED) and anhydrous potassium carbonate were purchased from Aladdin Co. LTD (China) were obtained from Sinopharm Chemical Reagent Co., Ltd (China). The black coating was purchased from Samhwa Paints Co., Ltd (China).

### Preparation of FPAE and the PFSF Film—Synthesis of Fluorinated poly(aryl ether) (FPAE)

2.34 g of decafluorobiphenyl, 2.40 g of 4,4′‐(hexafluoroisopropylidene) diphenol, 1.50 g of anhydrous potassium carbonate, and 15 mL of NMP were added to a 250 mL three‐necked flask equipped with magnetic stirring. The reaction was carried out at 80 °C for 12 h under a nitrogen atmosphere. The resulting brown viscous solution was poured into deionized water and a fibrous solid was obtained. After filtration and drying, the fibrous solid was re‐dissolved in dichloromethane and precipitated in methanol. The white precipitate, FPAE, was collected and dried at 60 °C under vacuum for 24 h.

### Preparation of FPAE and the PFSF Film—Preparation of PNIPAM Solution of Ethanol

60 mL of ethanol, 8.49 g of NIPAM, 0.39 g of BIS, and 0.41 g of AIBN were added to a 250 mL three‐necked flask equipped with magnetic stirring. The mixture was allowed to react at 70 °C for 4 h under a nitrogen atmosphere to obtain a PNIPAM in ethanol solution.

### Preparation of FPAE and the PFSF Film—Preparation of PFSF Film

FPAE was dissolved in NMP and cast onto a glass substrate. The FPAE‐coated glass substrate was then soaked in ethanol solution containing PNIPAM for 10 min (Figures S21–S25, Supporting Information). Then the composite film was immersed in aqueous 20 wt.% CaCl_2_ solution at 55 °C for 1h. The residual solvent was extracted and the PNIPAN in solution state was converted into a solid phase by raising the temperature to above 35 °C. Finally, the PFSF film was dried at room temperature.

### Preparation of FPAE and the PFSF Film—Preparation of PNIPAM Hydrogel

10 mg of BIS, 0.5 g of NIPAM, and 10 mg of KPS were added to 4.5 mL of deionized water with magnetic stirring. Then the mixture was purified in a nitrogen atmosphere with an ice bath for 30 min. Finally, 20 µL of TEMED was added to the precursor fluid to accelerate the gelation.

### Preparation of FPAE and the PFSF Film—Preparation of FPAE Radiative Cooling Film

FPAE was dissolved in NMP and cast onto a glass substrate. The FPAE‐coated glass substrate was then soaked in an ethanol bath for 10 min. Then the film was immersed in deionized water for 1 h, the residual solvent was extracted. Finally, the FPAE radiative cooling film was dried at room temperature.

## Conflict of Interest

The authors declare no conflict of interest.

## Supporting information



Supporting Information

## Data Availability

The datasets generated during and/or analysed during the current study are available from the corresponding author on reasonable request.

## References

[1] a) X. Meng , Z. Chen , C. Qian , Z. Song , L. Wang , Q. Li , X. Chen , ACS Appl. Mater. Interfaces 2022, 15, 2256; 36541618 10.1021/acsami.2c19422

[2] a) Y. Sun , H. He , X. Huang , Z. Guo , ACS Appl. Mater. Interfaces 2023, 15, 4799; 36635243 10.1021/acsami.2c18774

[3] a) Y. Tian , H. Shao , X. Liu , F. Chen , Y. Li , C. Tang , Y. Zheng , ACS Appl. Mater. Interfaces 2021, 13, 22521; 33950669 10.1021/acsami.1c04046

[4] a) C. Lin , Y. Li , C. Chi , Y. S. Kwon , J. Huang , Z. Wu , J. Zheng , G. Liu , C. Y. Tso , C. Y. H. Chao , B. Huang , Adv. Mater. 2022, 34, 09350; 10.1002/adma.202109350 35038775

[5] a) C.‐H. Xue , R.‐X. Wei , X.‐J. Guo , B.‐Y. Liu , M.‐M. Du , M.‐C. Huang , H.‐G. Li , S.‐T. Jia , Compos. Sci. Technol. 2022, 220, 109279;

[6] a) M. Xiong , Y. Sheng , Y. Di , F. Xing , L. Yu , J. Zhang , W. Zhou , C. Liu , L. Dong , Z. Gan , ACS Appl. Mater. Interfaces 2021, 13, 33566; 34240841 10.1021/acsami.1c09533

[7] a) X. Cai , Y. Wang , Y. Luo , J. Xu , L. Zhao , Y. Lin , Y. Ning , J. Wang , L. Gao , D. Li , ACS Appl. Mater. Interfaces 2022, 14, 27222; 35657958 10.1021/acsami.2c05943

[8] a) C. Fan , Y. Zhang , Z. Long , A. Mensah , Q. Wang , P. Lv , Q. Wei , Adv. Funct. Mater. 2023, 33, 00794;

[9] a) Q. Zhang , Y. Lv , Y. Wang , S. Yu , C. Li , R. Ma , Y. Chen , Nat. Commun. 2022, 13, 32528; 10.1038/s41467-022-32528-1 PMC9391366 35985989

[10] a) C. Zhang , J. Yang , Y. Li , J. Song , J. Guo , Y. Fang , X. Yang , Q. Yang , D. Wang , X. Deng , Adv. Funct. Mater. 2022, 32, 08144;

[11] a) M. Liu , X. Li , L. Li , L. Li , S. Zhao , K. Lu , K. Chen , J. Zhu , T. Zhou , C. Hu , Z. Lin , C. Xu , B. Zhao , G. Zhang , G. Pei , C. Zou , ACS Nano 2023, 17, 9501; 37166276 10.1021/acsnano.3c01755

[12] K. Lv , Y. Zhu , L. Wang , Z. Chen , Z. Zhang , Y. Gao , Ceram. Int. 2023, 49, 7387.

[13] a) J. Wang , D. Yuan , P. Hu , Y. Wang , J. Wang , Q. Li , Adv. Funct. Mater. 2023, 33, 00441;

[14] S. Chen , G. Jiang , J. Zhou , G. Wang , Y. Zhu , W. Cheng , G. Xu , D. Zhao , H. Yu , Adv. Funct. Mater. 2023, 33, 14382.

[15] W. Wei , Y. Zhu , Q. Li , Z. Cheng , Y. Yao , Q. Zhao , P. Zhang , X. Liu , Z. Chen , F. Xu , Y. Gao , Sol. Energy Mater. Sol. Cells 2020, 211, 110525.

[16] a) S. Wang , Y. Zhou , T. Jiang , R. Yang , G. Tan , Y. Long , Nano Energy 2021, 89, 106440;

[17] a) J. Liu , H. Tang , C. Jiang , S. Wu , L. Ye , D. Zhao , Z. Zhou , Adv. Funct. Mater. 2022, 32, 06962;

[18] a) X. Li , B. Sun , C. Sui , A. Nandi , H. Fang , Y. Peng , G. Tan , P.‐C. Hsu , Nat. Commun. 2020, 11, 19790; 10.1038/s41467-020-19790-x PMC7705009 33257693

[19] a) L. Qi , L. Chen , W. Cai , C. Wang , B. Wang , Y. Hu , W. Xing , Chem. Eng. J. 2023, 475, 146272;

[20] J. Mandal , M. Jia , A. Overvig , Y. Fu , E. Che , N. Yu , Y. Yang , Joule 2019, 3, 3088.

[21] X. Li , Z. Ding , G. E. Lio , J. Zhao , H. Xu , L. Pattelli , L. Pan , Y. Li , Chem. Eng. J. 2023, 461, 142095.

[22] a) J. M. C. Puguan , P. V. Rathod , H. Kim , ACS Appl. Mater. Interfaces 2021, 13, 36330; 34308626 10.1021/acsami.1c09561

[23] a) X. Mei , T. Wang , M. Chen , L. Wu , J. Mater. Chem. A. 2022, 10, 11092;

[24] a) Z. Yu , Y. Ma , L. Mao , Y. Lian , Y. Xiao , Z. Chen , Y. Zhang , Mater. Horiz. 2024, 11, 207; 37888540 10.1039/d3mh01376f

[25] M. Yang , H. Zhong , T. Li , B. Wu , Z. Wang , D. Sun , ACS Nano 2023, 17, 1693. 10.1021/acsnano.2c11916 36633491

