# Supplementary material for: Self‐Adaptive Reflectance Film for Passive Temperature Regulation in Diverse Environments
Source: Adv Sci (Weinh). 2025 Apr 26;12(28):2415121. doi: 10.1002/advs.202415121 (PMC12302567; doi:10.1002/advs.202415121)
Supplement: Supplementary file 1 — Supporting Information [file ADVS-12-2415121-s001.docx]

**Supporting Information**

**Self-adaptive Reflectance Film for Passive Temperature Regulation in Diverse Environment**

*Shuo Yang^a^, Sizhe Tang^a^, Yufeng Cai^b^, Zhiming Ye^a^, Xiangbin Zou^a^, Xueyu Yuan^a^, Yujie Song^c^, Bing Li*^a^, Dongyan Tang*^a, d^, Ming Liu*^a, c^*

*a. School of Chemistry and Chemical Engineering, Harbin Institute of Technology, Harbin, 150001, PR China*

*b. State Key Laboratory of Special Functional Waterproof Materials, Beijing Oriental Yuhong Waterproof Technology Co. Ltd，Beijing 101111, China*

*c. Engineering Laboratory of Advanced Energy Materials, Ningbo Institute of Materials Technology & Engineering, Chinese Academy of Sciences, Ningbo 315201, China*

*d. State Key Laboratory of Advanced Welding and Joining, Harbin Institute of Technology，Harbin 150001*

*Corresponding authors

*E-mail address*: bing.li2020@hit.edu.cn (Bing Li)

dytang@hit.edu.cn (Dongyan Tang)

liuming0117@hit.edu.cn (Ming Liu)

**List of Figures and Tables:**

**Supplementary Figure 1.** The UL94 combustion test.

**Supplementary Figure 2.** SEM images of the FPAE film and the PFSF film surface.

**Supplementary Figure 3.** SEM image of the cross section of the FPAE film.

**Supplementary Figure 4.** The reflectance spectra of FPAE film.

**Supplementary Figure 5.** TGA and DTGA curves of PFSF and FPAE film.

**Supplementary Figure 6.** The reflectance spectra of the PFSF film stored at 20 ℃ and 95% - 35% RH different time.

**Supplementary Figure 7.** The reflectance spectra of the PFSF film stored at 30 ℃ and 95% - 35% RH different time.

**Supplementary Figure 8.** The reflectance spectra of the PFSF film stored at 40 ℃ and 95% - 35% RH different time.

**Supplementary Figure 9.** The reflectance spectra of the PFSF film stored at 50 ℃ and 95% - 35% RH different time.

**Supplementary Figure 10.** The average reflectance of PFSF films stored in different environments for 15 mins.

**Supplementary Figure 11.** Water uptaken of PFSF membrane in different environments for 60 mins.

**Supplementary Figure 12.** G’ and G” as a function of temperature for PNIPAM.

**Supplementary Figure 13.** Digital image of PNIPAM under different Temperature.

**Supplementary Figure 14.** The reflectance spectra of PFSF and PNIPAM in 40 ^o^C.

**Supplementary Figure 15.** Digital image of the cooling test apparatus in Fuzhou.

**Supplementary Figure 16.** Thermal infrared image of PFSF film on cement pavement, palm, and bush.

**Supplementary Figure 17.** The temperature difference between the bare surface and PFSF covered surfaces.

**Supplementary Figure 18.** Schematic of the model building for energy saving simulation.

**Supplementary Figure 19.** Schematic of PFSF film temperature adjust process during one day.

**Supplementary Figure 20.** The film can be folded, crimped, and can be cut into different shapes.

**Supplementary Figure 21.** Thermal infrared image of moist and dry states of PFSF and PFSF-B film under one sun irradiation.

**Supplementary Figure 22.** Temperature variation of moist and dry states of PFSF film and PFSF-B film.

**Supplementary Figure 23.** SEM image of the cross section of the PFSF film with 1 min solvent exchange time.

**Supplementary Figure 24.** SEM image of the cross section of the PFSF film with 5 mins solvent exchange time.

**Supplementary Figure 25.** SEM image of the cross section of the PFSF film with 15 mins solvent exchange time.

**Supplementary Table 1.** The fire retardant test results.

**Supplementary Table 2.** Thermal properties of the PFSF film used in Energyplus simulation.

**Table of Contents:**

Supplementary Note 1. Thermal properties of FPAE and PFSF film

Supplementary Note 2: Rheological properties of PNIPAM hydrogel

Supplementary Note 3: The temperature change of PFSF film in outdoor test

Supplementary Note 4: The building modified of PFSF films

Supplementary Note 5: The temperature change of PFSF-B film in indoor test

Supplementary note 6: The effects of solvent exchange time on the cross-section morphologies

**1. Characterization**

***Films characterization:***

FT-IR spectra were recorded on a Thermo Nicolet 5700 FT-IR spectrometer using the transmission mode.

***Morphological characterization:***

The surface morphologies of the gold sputter-coated PFSF films were obtained by scanning electron microscope (SEM) under an acceleration voltage of 15 kV.

***Optical characterization:***

The reflectivity spectra in the ultraviolet-visible-near infrared (200nm – 2500nm) was measured by spectrophotometer (Lambda – 950, Perlinelmer) with an integrating sphere and BaSO_4_ as the baseline reference. Fourier transforms infrared spectrometer with a gold integrating sphere was used to measure the mid-infrared range (4 μm – 25 μm). Before test, the sample was placed in the set constant temperature and humidity box.

***Solar reflectance and mid-infrared emittance calculation***

The average reflectance is calculated by:

$r=\frac{\int_{0.3\mu m}^{2.5\mu m} d\lambda R(\lambda)E_{solar}(\lambda)}{\int_{0.3\mu m}^{2.5\mu m} {d\lambda E}_{solar}(\lambda)}$ (Equation S1)

Where R(λ) is the measured reflectance spectrum of the test sample, E_solar_ (λ) is the solar irradiance spectra (AM 1.5)

The average mid-infrared emittance is calculated by:

$e=\frac{\int_{8\mu m}^{13\mu m} d\lambda E_{BB}(T, \lambda)E(\lambda)}{\int_{8\mu m}^{13\mu m} d\lambda E_{BB}(T,\lambda)}$ (Equation S2)

Where E_BB_ (T, λ) is the theoretical blackbody spectral radiance, E (λ) is the experimentally measured emittance of the test sample.

***Surface wettability characterization:***

The water contact angle (WCA) was measured by a Theta contact angle tester (Biolin Scientific). Water droplets of 5 µm were used, and the WCA was an average of data obtained at different positions of the film surface.

***Calculation of enthalpy of evaporatio***n

By keeping the input power (Uin) for water evaporation constant, we can calculate the equivalent evaporation enthalpy (Eequ) of water in hydrogels using the following equation:

$Uin=E0*R0=Rh*Eequ$ (Equation S3)

In this equation, R h indicates the evaporation rate of water in hydrogel in the dark, R0 denotes the evaporation rate of water under dark evaporation conditions, and E0 represents the theoretical evaporation enthalpy of water (2450 J g^-1^)

***Fire retardant test***

Prior to the test, the sample was placed at 20 °C, 50 % RH for 48 hours. The sample was clamped vertically leaving 6 mm from the top free end, the lower end of the sample is 300 mm above the horizontal surface layer. A blowtorch is used to ignite the sample from the bottom free end for two 10 s intervals separated by the time it takes for flaming combustion to cease after the first ignition. A total of 5 samples were tested. The grade of the samples was evaluated according to the grading criterion.


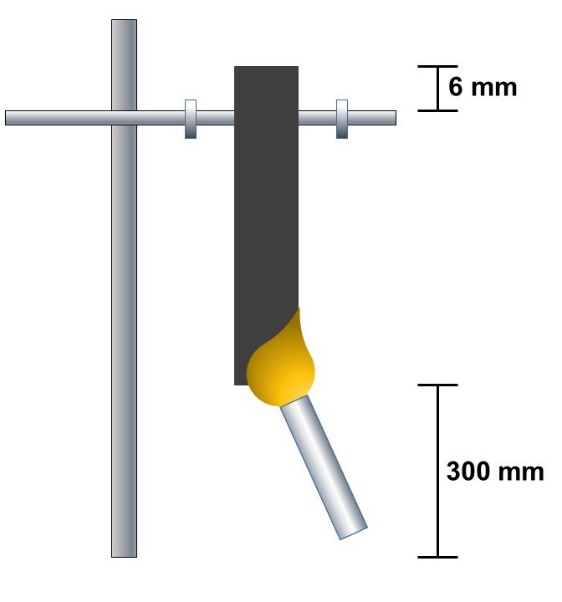


**Supplementary Figure 1.** The UL94 combustion test

***Radiative cooling demonstration:***

The indoor cooling effect was demonstrated by using an analog solar xenon lamp system to cast uniform power irradiation (300 mw/cm^2^) on the surface of a sample. A polystyrene box with an internal dimension of 30.5 cm × 18.5 cm × 14.5 cm was used as a model room to investigate the cooling capacity of the film. A square opening of 10 cm × 10 cm was cut out at the top and covered with the sample to be tested. The box was wrapped in aluminum foil to reflect the sun’s radiation and the heat from the surrounding environment. The outermost layer of the box was covered with polyethylene film to isolate the influence of external air through convection. Digital thermocouples were placed inside and outside the box to monitor the temperature fluctuations during the test. The ambient temperature was recorded concurrently as a reference. In order to evaluate the radiation cooling effectiveness of the film, two strategies to minimize the effect of heat conduction via conduction and convection. The foam box isolates the conduction heat transfer, and the PE film wrapped in the outer layer isolates the convection heat transfer in the air.

The same setup was placed outdoor to better simulate the cooling effect during the actual application. The test location is Harbin city, Heilongjiang Province, China (45°4’N, 126°37’E) and Fuzhou city, Fujian Province, China (26°4’N, 119°20’E). Three identical setups were placed in a square to avoid blockage from the surrounding buildings and trees. The opening on the setup was covered by glass coated with PFSF film and FPAE film. An uncovered setup, marked as ambient, was used as a reference.

The PNIPAM precipitates deposited on the surface of the FPAE film formed a dense layer covering the surface of the FPAE film (Supplementary Fig.2, 3).


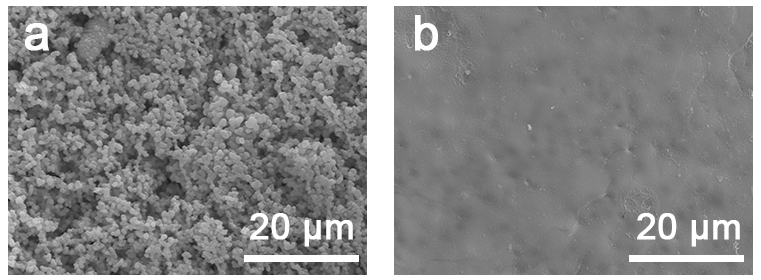


**Supplementary Figure 2.** SEM images of (a) the FPAE film and (b) the PFSF film surface.


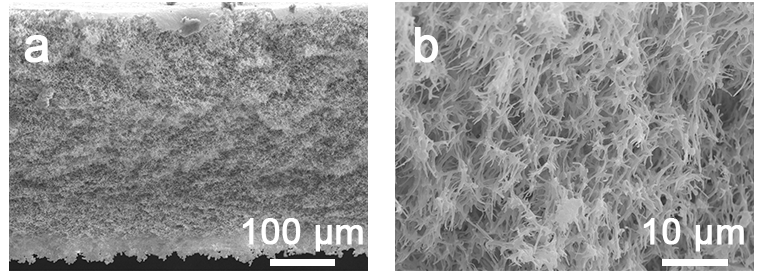


**Supplementary Figure 3.** SEM image of (a) the cross section of the FPAE film, and (b) the magnification of cross section of the FPAE film.

The FPAE film shown an average reflectance of 96.2% (Supplementary Figure. 4, Equation S1), a combined effect from the polymer microspheres and the air pockets within the film.


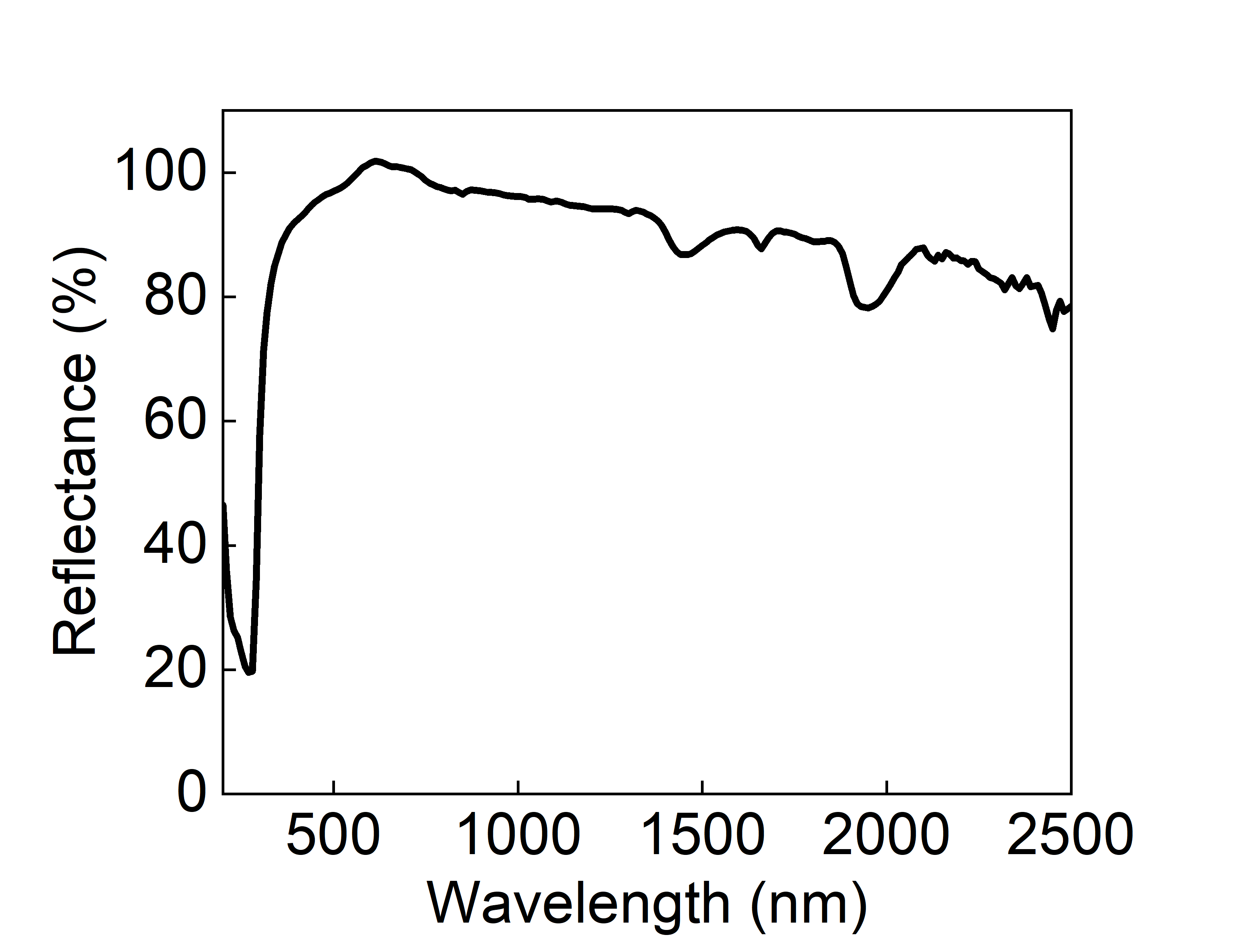


**Supplementary Figure 4.** The reflectance spectra of FPAE film.

**Supplementary Note 1: Thermal properties of FPAE and PFSF film**

Supplementary Figure 5 shows the thermal degradation process of PFSF. It can be seen from the figure that when the temperature rises to 143 ^o^C, the decomposed mass of the PFSF film in the first stage is 26.7%, while the pyrolysis of FPAE has not yet begun, indicating that the weight of pyrolysis at this time is PNIPAM. Then, the PFSF and FPAE begin to have obvious pyrolysis at 470 ^o^C. At this time, the pyrolysis part of PFSF film is FPAE.

**
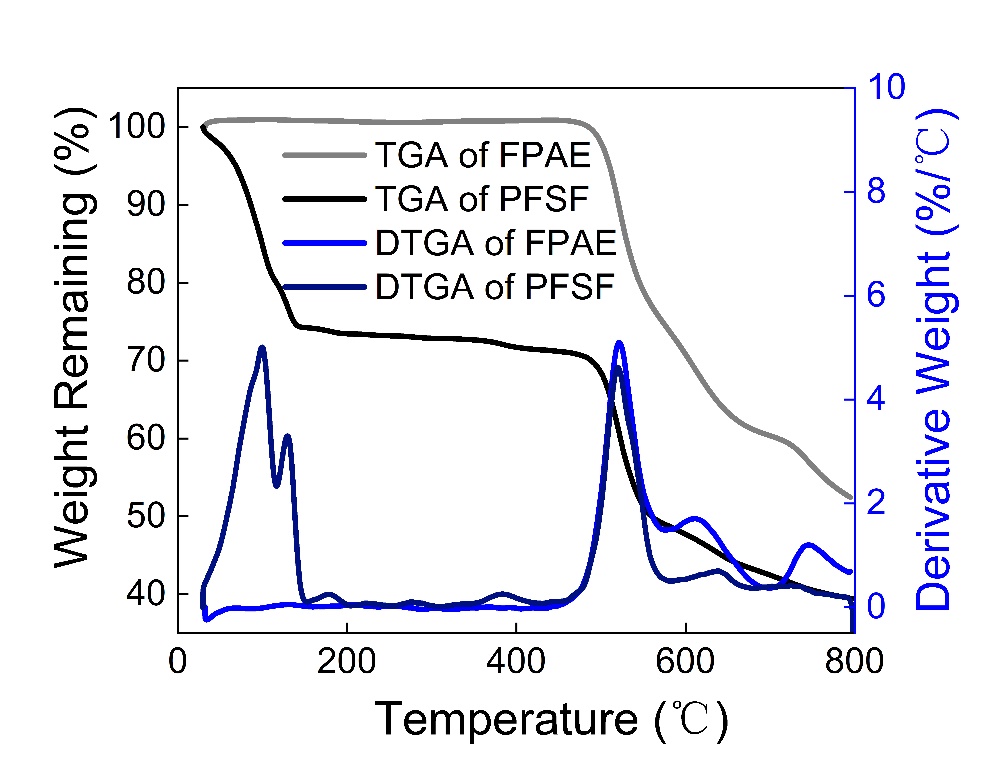
**

**Supplementary Figure 5.** TGA and DTGA curves of PFSF and FPAE film.


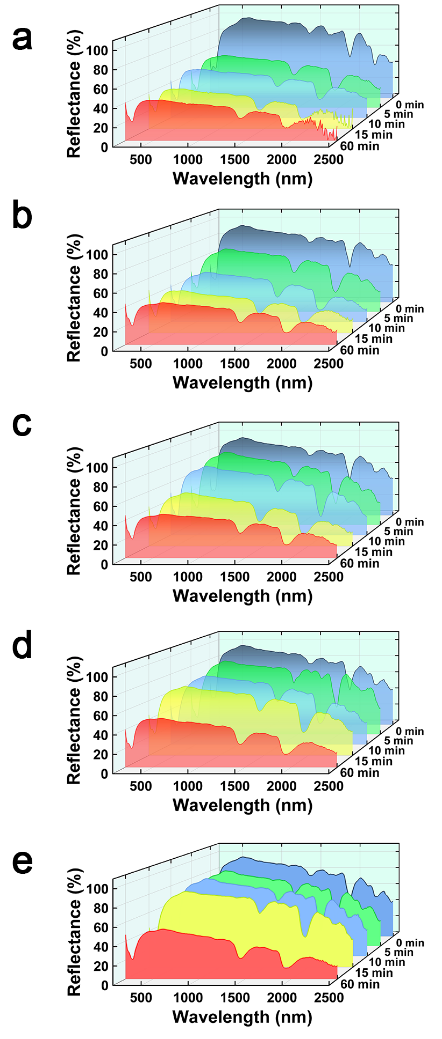


**Supplementary Figure 6.** The reflectance spectra of the PFSF film stored at 20 ℃ and (a) 95% RH, (b) 80% RH, (c) 65% RH, (d) 50% RH, and 35% RH different time.


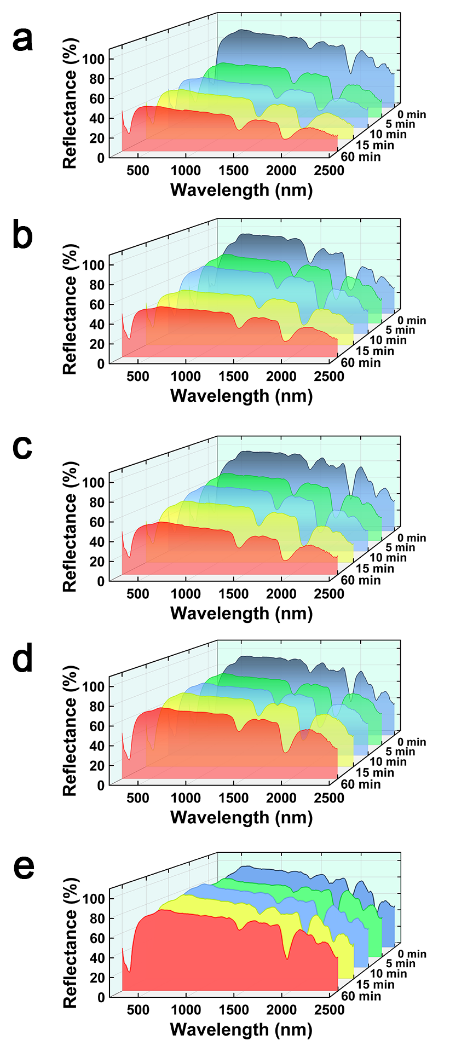


**Supplementary Figure 7.** The reflectance spectra of the PFSF film stored at 30 ℃ and (a) 95% RH, (b) 80% RH, (c) 65% RH, (d) 50% RH, and 35% RH different time.


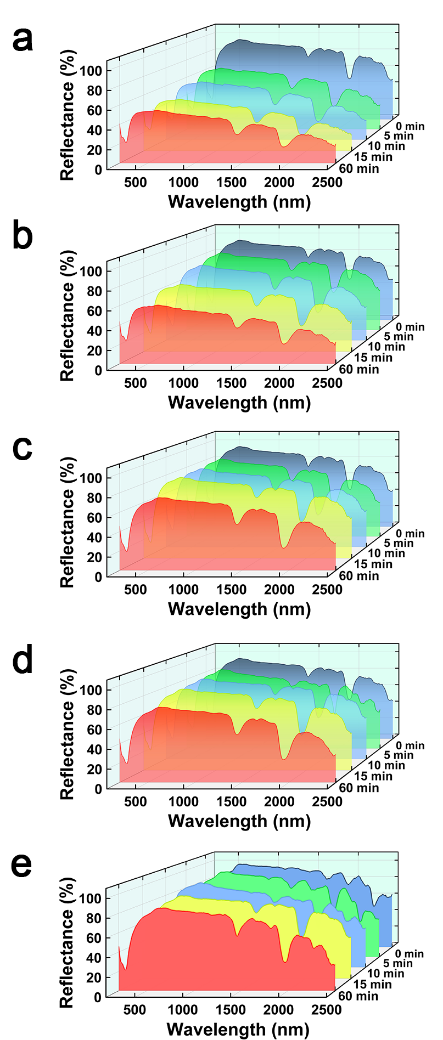


**Supplementary Figure 8.** The reflectance spectra of the PFSF film stored at 40 ℃ and (a) 95% RH, (b) 80% RH, (c) 65% RH, (d) 50% RH, and 35% RH different time.


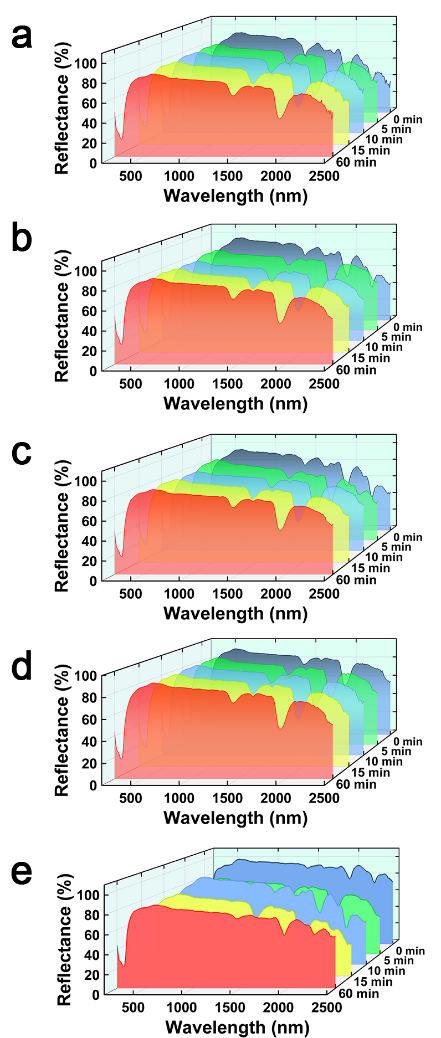


**Supplementary Figure 9.** The reflectance spectra of the PFSF film stored at 50 ℃ and (a) 95% RH, (b) 80% RH, (c) 65% RH, (d) 50% RH, and 35% RH different time.


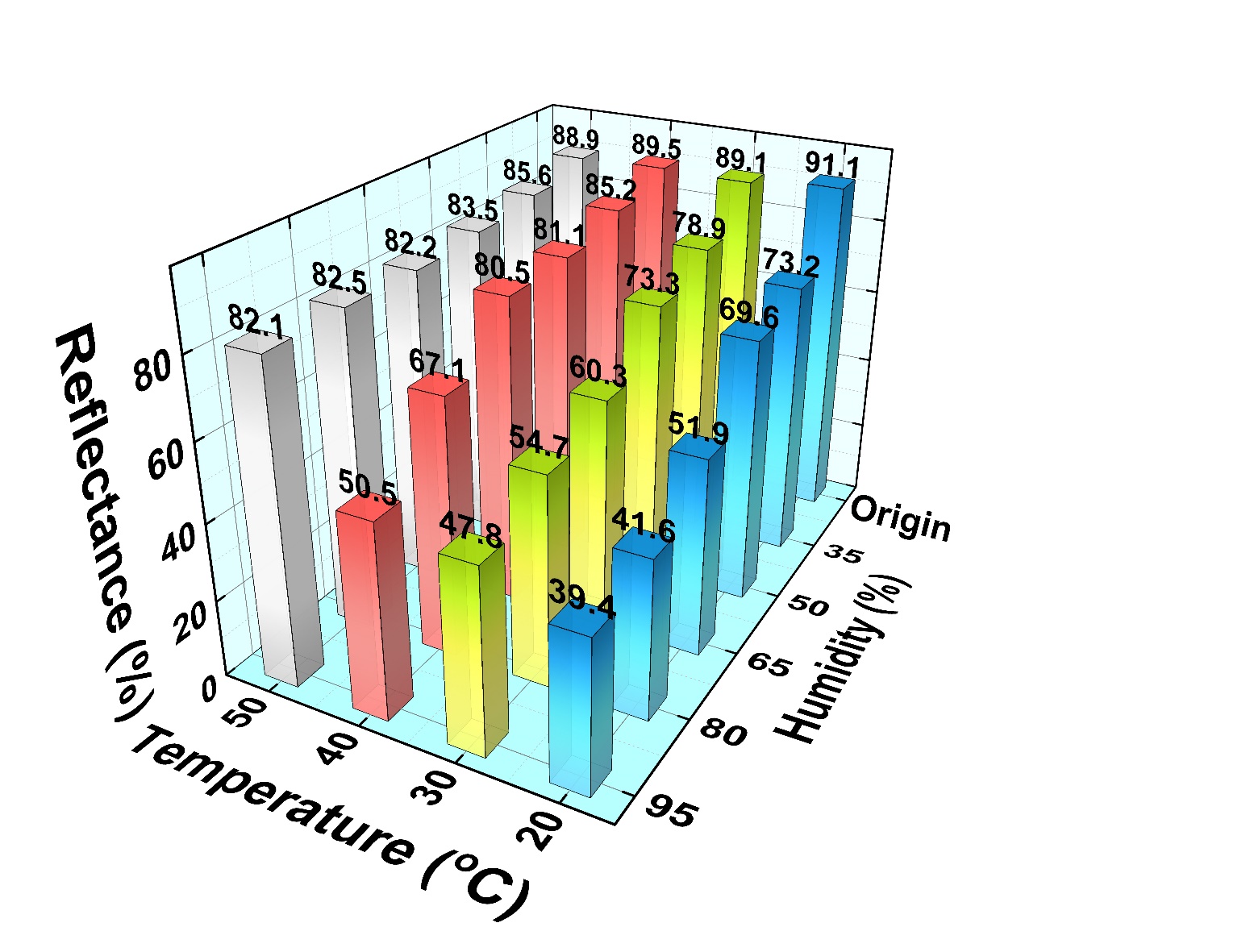


**Supplementary Figure 10.** The average reflectance of PFSF films stored in different environments for 15 mins.


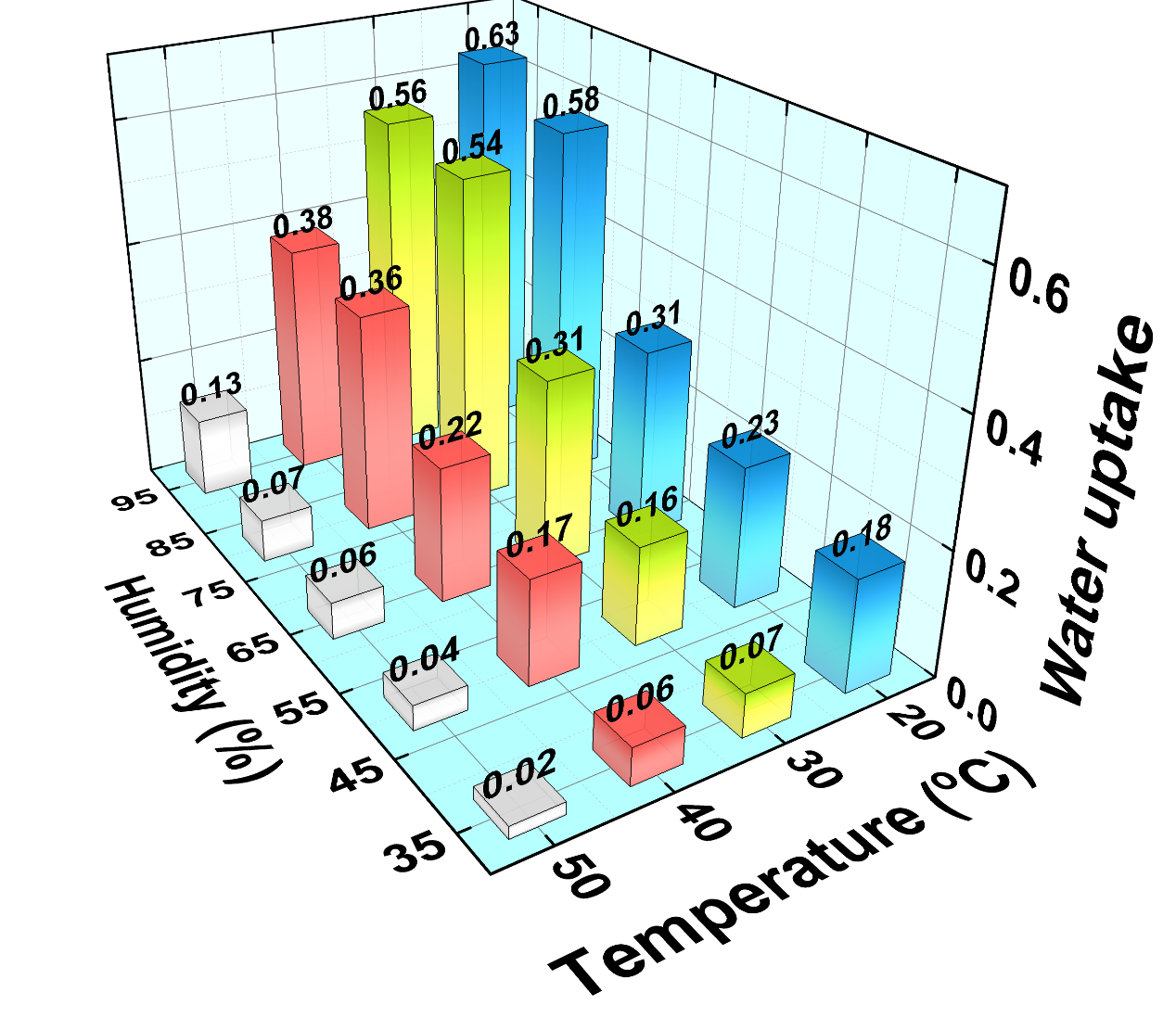


**Supplementary Figure 11.** Water uptaken of PFSF membrane in different environments for 60 mins.

**Supplementary Note 2: Rheological properties of PNIPAM hydrogel**

The energy storage modulus (G’) and dissipation modulus (G”) of PNIPAM hydrogel were measured by rheometer. It can be seen from Supplementary Figure 12 that both G’ and G” begin to rise when the temperature rises to 32 ^o^C. This is because when the temperature reaches the LCST of hydrogel, the hydrogen bond between PNIPAM molecular chain and water molecules begins to break, PNIPAM forms intramolecular hydrogen bond, and the gel modulus rises accordingly. Therefore, the LCST of PNIPAM hydrogel is 32 ^o^C.


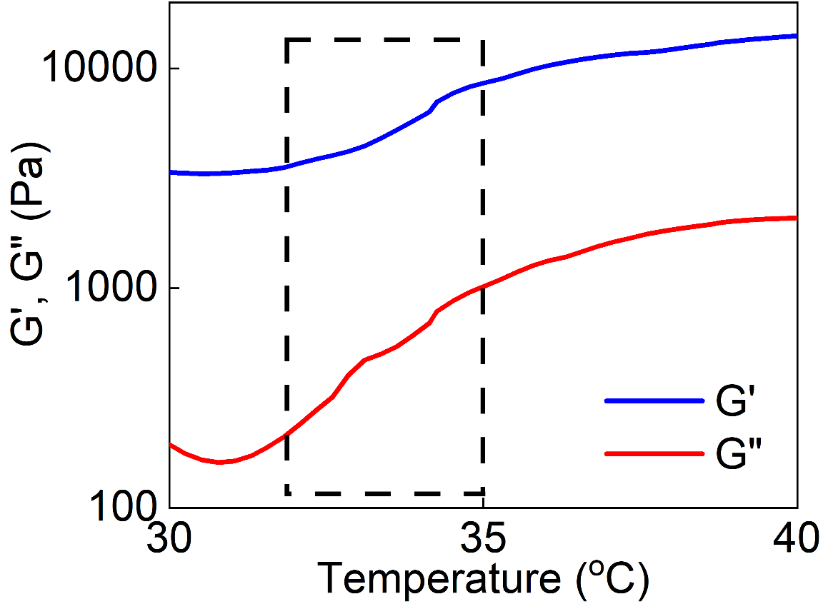


**Supplementary Figure 12.** G’ and G” as a function of temperature for PNIPAM.

PNIPAM is a commonly used temperature-responsive hydrogel for smart window applications for its near room temperature low critical solution temperature (LCST) (Supplementary Fig. 12). Under LCST, PNIPAM is a hydrophilic state with a transparent appearance. When the temperature rises, the internal molecules of PNIPAM flip over, the hydrogen bond with water breaks, the polymer changes to a hydrophobic state, and the hydrogel split phase become white and opaque (Supplement Fig. 13).


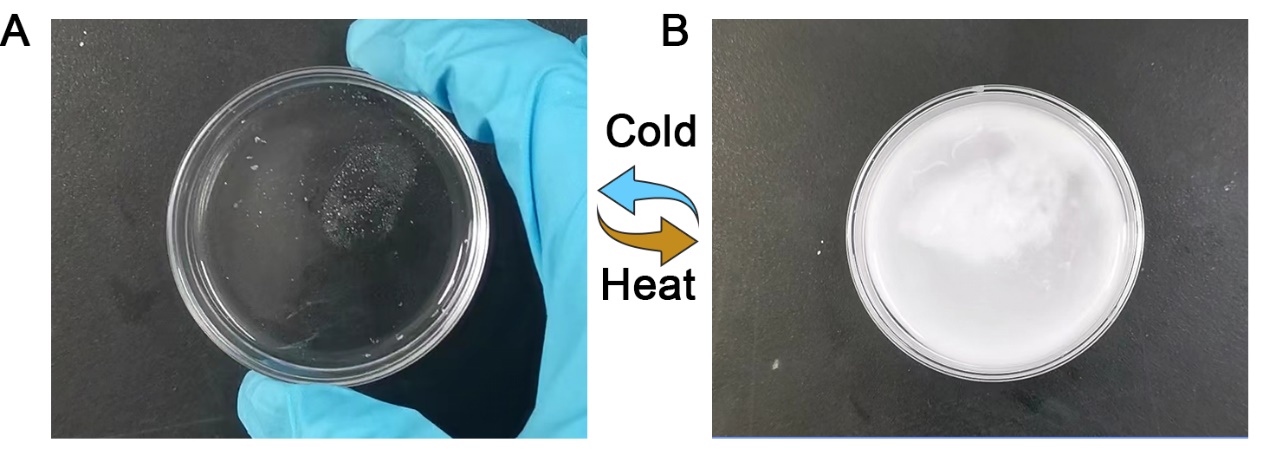


**Supplementary Figure 13.** Digital image of PNIPAM (A) under LCST (Lower Critical Solution Temperature) (20 ^o^C) and (B) exceed LCST (40 ^o^C).

The application of PNIPAM hydrogel is often realized with encapsulation to address the fragile nature and the need of a moisture-rich environment. Hence leakage and weight became one of the bottlenecks for the practical applications of such systems. On the contrary, the PFSF film in this work exploits the temperature-dependent hygroscopicity of the CaCl_2_ loaded PNIPAM without the mentioned hassles. Supplement Fig. 14 shows that the average reflectance of a 6 mm thick PNIPAM hydrogel above LCST and a PFSF film of thickness 0.38 mm are 72.1%, and 90.1%, respectively. The density of PFSF film is 0.197 Kg/m^2^ in the dry state and 0.320 Kg/m^2^ after moisture absorption whereas the density of swelled PNIPAM hydrogel is 15.2 Kg/m^2^. PFSF film is only 1/48 of the weight of PNIPAM hydrogel.


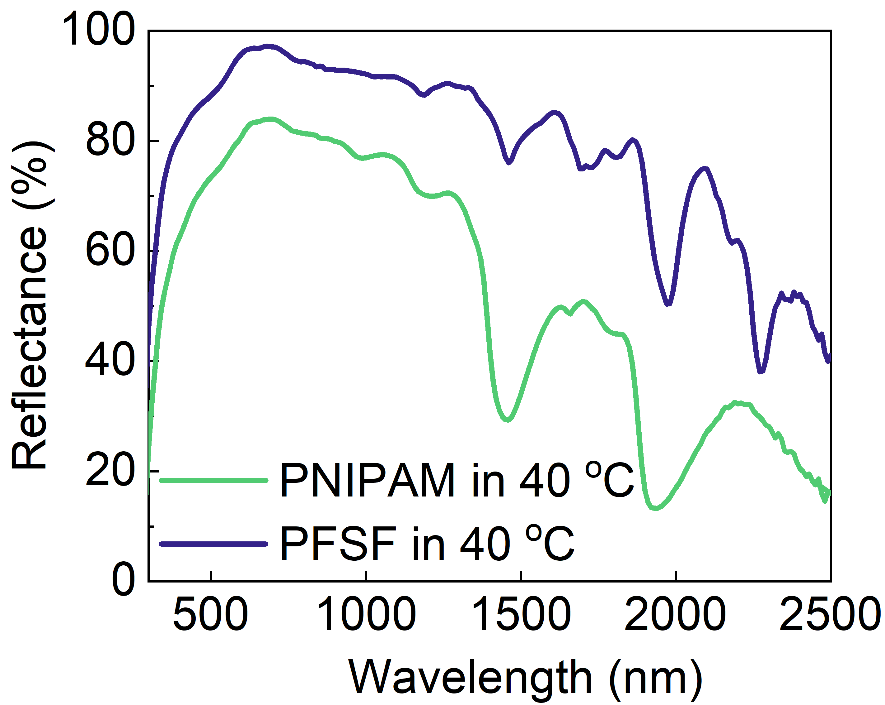


**Supplementary Figure 14.** The reflectance spectra of PFSF and PNIPAM in 40 ^o^C.


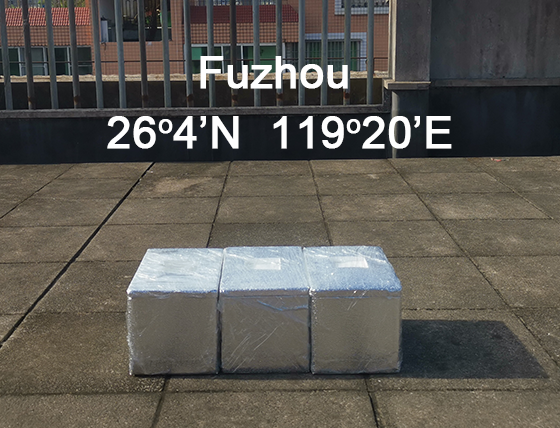


**Supplementary Figure 15.** Digital image of the cooling test apparatus placed on a roof in Fuzhou.

**Supplementary Note 3: The temperature change of PFSF film in outdoor**

The PFSF film was placed on the cement pavement in direct sunlight, and thermal infrared images of the PFSF were taken every 15 minutes with a thermal infrared camera. It can be seen from Supplement Fig. 16A that the temperature (∆T_1_) of the PFSF is 8.8℃ lower than that of the road surface at the beginning. After 60 minutes of irradiation, the temperature of the PFSF is still 7.8 ℃ lower than that of the road surface (Supplement Fig. 17A). The PFSF film also was placed in the palm and then thermal infrared images were taken every five minutes outside in direct sunlight (Supplement Fig. 16B). As can be seen from Supplement Fig. 17Ba, the initial temperature of PFSF is 6.4 ℃ lower than that of the palm, and after 20 minutes of sunlight exposure, ∆T_2_ still reach 5.0 ℃. This shows that PFSF film has the potential to be applied to outdoor human cooling protection. The PFSF was also placed on the bush, taking thermal infrared images every 15 minutes. It can be seen from Supplement Fig. 16C that the temperature of the PFSF is similar to that of shrubs, and the outline of the PFSF in the thermal infrared image is fuzzy. Even after 60 minutes of sunlight exposure, the temperature (∆T_3_) of the PFSF film placed on the surface of the shrub was still 19.2℃ lower than that of the road surface (Supplement Fig. 17C). The above experiments show that PFSF can effectively prevent the rise of surface temperature under the conditions of direct sunlight, and has the prospect of application in the temperature protection of buildings, vehicles and human bodies.


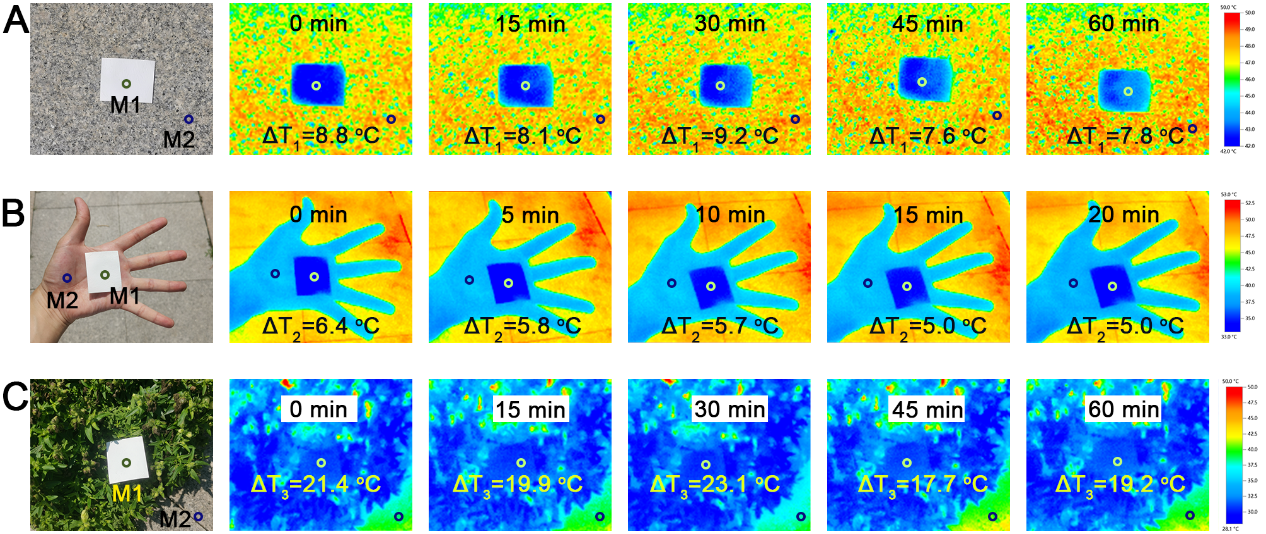


**Supplementary Figure 16.** (A) Thermal infrared image of PFSF film on cement pavement; (B) palm; (C) and bush. ΔT_x_ is the temperature difference between the temperature recorded at location M1 and M2.


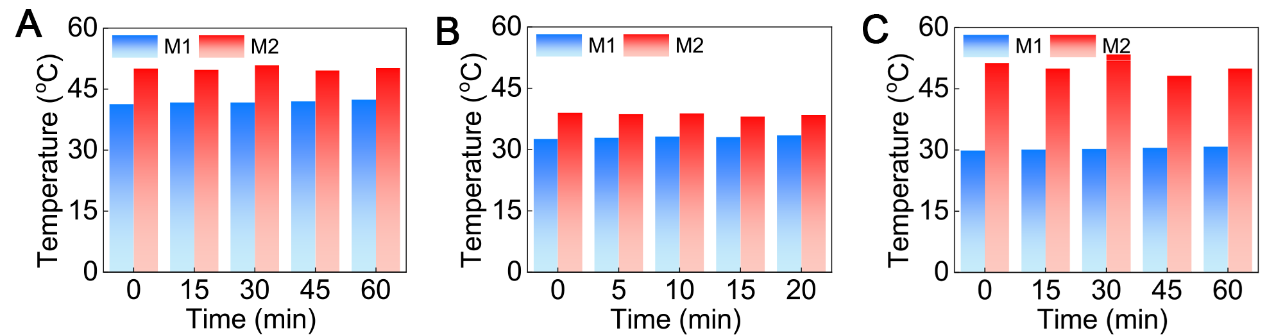


**Supplementary Figure 17.** The temperature difference between the bare surface and PFSF covered surfaces of (A) cement pavement, (B) palm, and (C) bush.

**Supplementary Note 4:** **The building modified of PFSF films**

EnergyPlus simulation is used to assess the energy consumption of PFSF-covered buildings for temperature regulation over the course of a year. A building model was established (15.2 × 30.5 × 3.0 m) by software. According to Lambertian emission properties, the roof surface of the building was covered with PFSF films (Supplementary Figure 18). The properties of the films are shown in Supplementary Table2. Typical meteorological years in fifteen cities across China were selected for the simulation of energy saving effects.


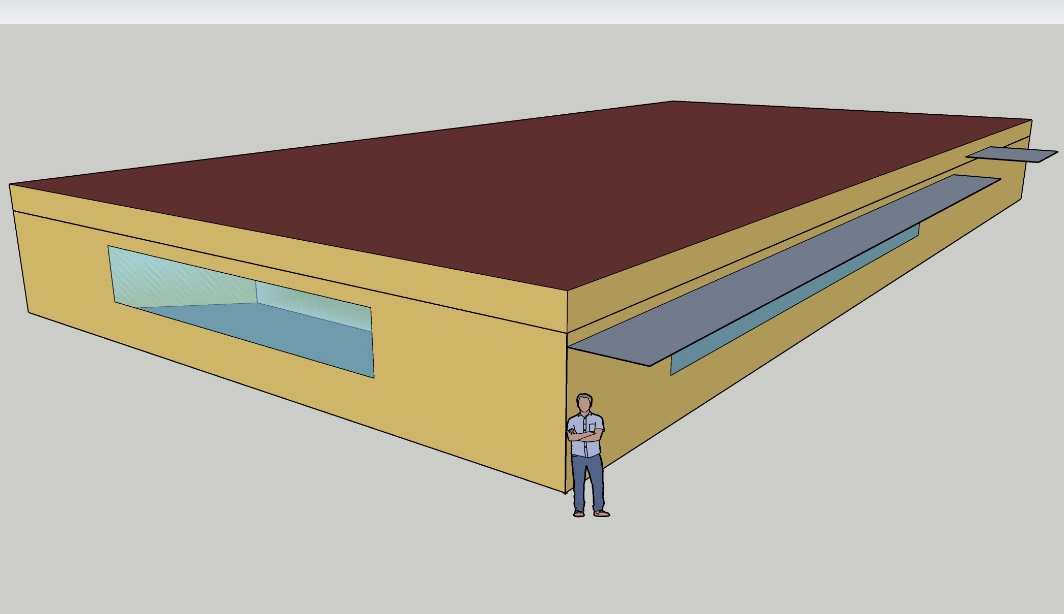


**Supplementary Figure 18.** Schematic of the model building for energy saving simulation.


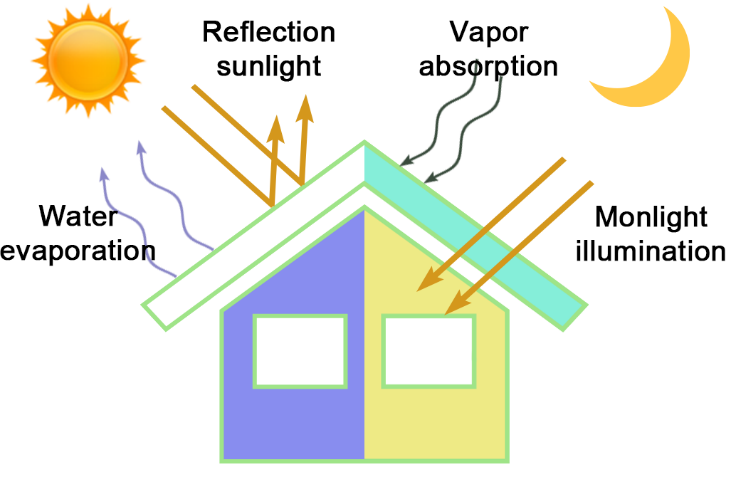


**Supplementary Figure 19.** Schematic of PFSF film temperature adjust process during one day.


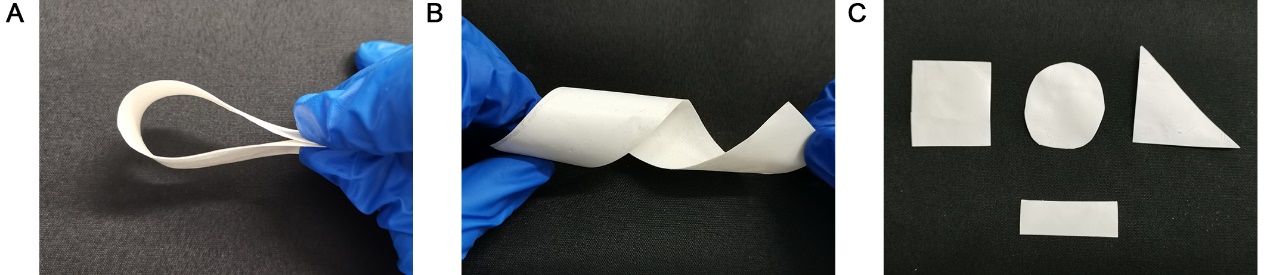


**Supplementary Figure 20.** The film can be (A) folded and (B) crimped, and (C) can be cut into different shapes.

**Supplementary Note 5: The temperature change of PFSF-B film in indoor test**

A high power xenon lamp is used to simulate solar radiation, and the surface of PFSF and PFSF-B film are illuminated with uniform light for the same amount of time. Then the surface temperatures of PFSF and PFSF-B films were measured by infrared thermometers at 1, 3, 5, 7 and 10 min, respectively. As can be seen from Supplement Fig. 21A, the heating rate of PFSF-B in hygroscopic state is significantly higher than that of PFSF film, and according to Supplement Fig. 22A the ∆T_4_ can reach 13.2℃ after heating for 10 min. In the dry state, PFSF and PFSF-B have similar temperatures, and the ∆T_5_ is only 1.4℃ after heating for 10 min (Supplement Fig. 21B, 22B). It is proved that PFSF-B film has similar reflectance and radiation cooling effect to PFSF film in dry state, and PFSF-B film has excellent photothermal conversion performance after moisture absorption, and can convert solar radiation into heat energy. By simply applying an endothermic coating to the back of the PFSF film, PFSF-B film with both cooling and heating functions can be obtained.


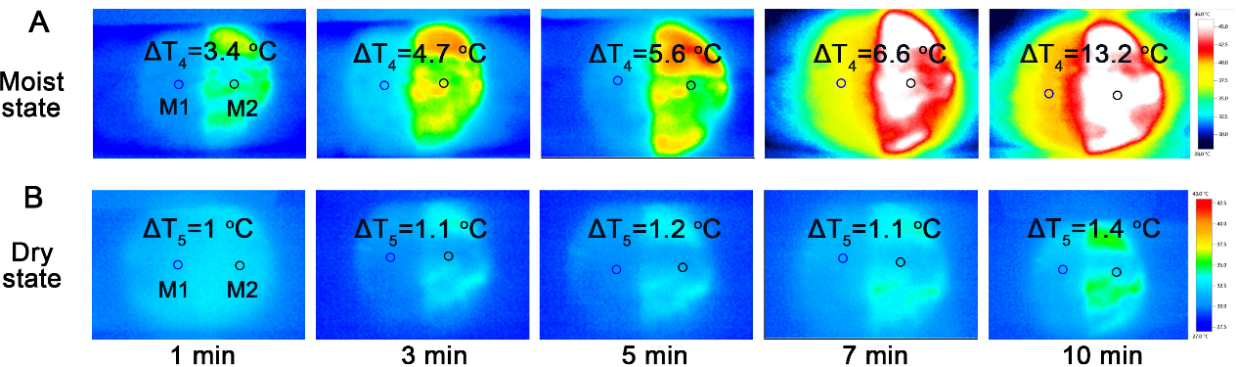


**Supplementary Figure 21.** Thermal infrared image of moist (A) and dry (B) states of PFSF film (left) and PFSF-B film (right) under one sun irradiation.


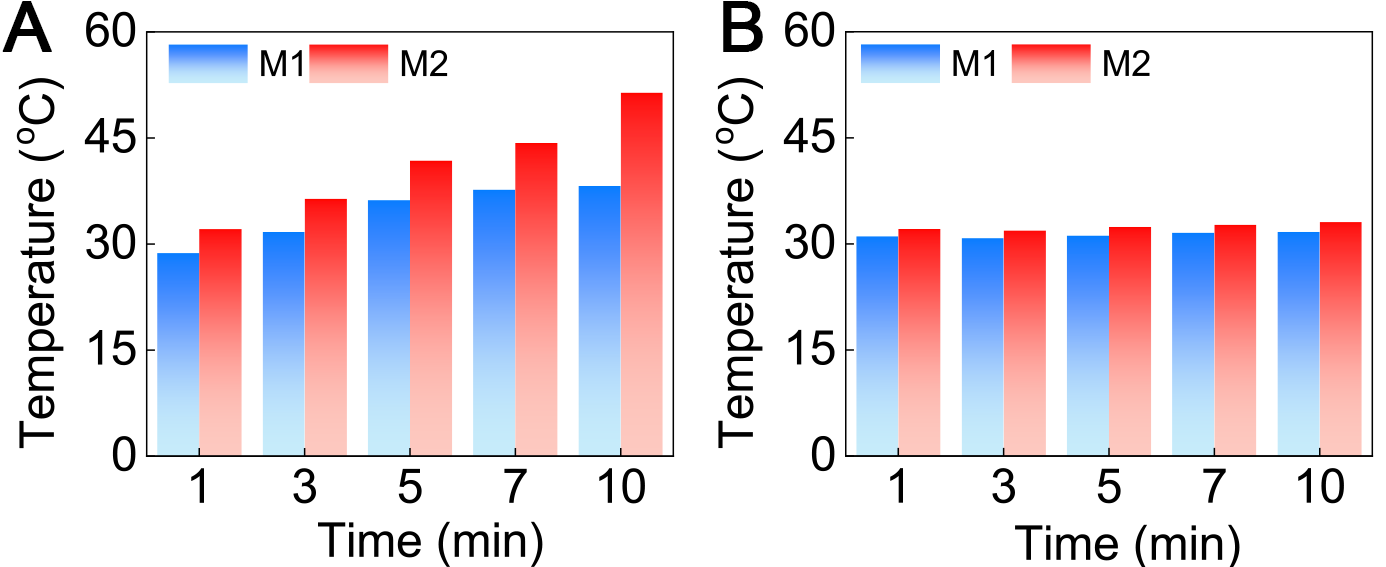


**Supplementary Figure 22.** Temperature variation of moist (A) and dry (B) states of PFSF film and PFSF-B film.

**Supplementary note 6: The effects of solvent exchange time on the cross section morphologies**

The effects of solvent exchange time were studied by immersing the film in PNIPAM solution for 1, 5, 10, and 15 mins prior to morphological examination. Because the exchange time in the PNIPAM solution is too short, the fast exchange rate between NMP and water causes the cross section of the film to form wormlike channels, and such cross section structure cannot be effectively filled by PNIPAM after water absorption, so it loses the ability of reflectivity regulation (Supplementary Figure 25).


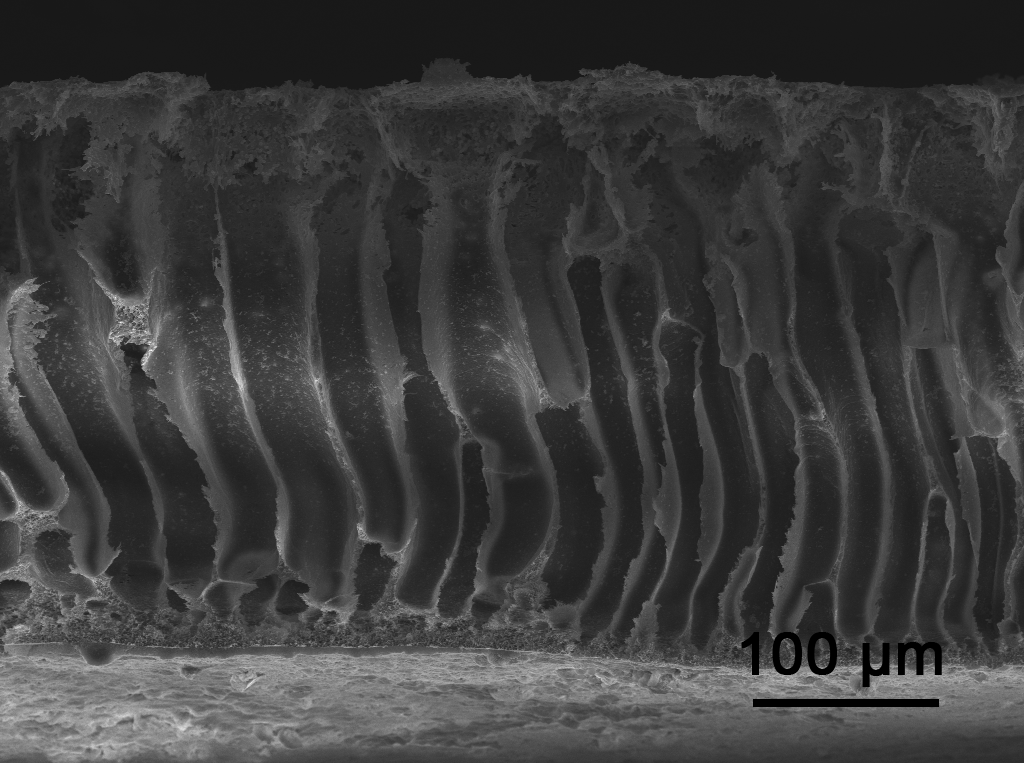


**Supplementary Figure 23.** SEM image of the cross section of the PFSF film with 1 min solvent exchange time in PNIPAM solution.

As the solvent exchange time increased to 5 minutes, the film section began to form a micrometer-scale porous structure, but due to insufficient exchange time with the PNIPAM solution, PNIPAM and FPAE could not form a relatively tight interpenetrating structure (Supplementary Figure 26).


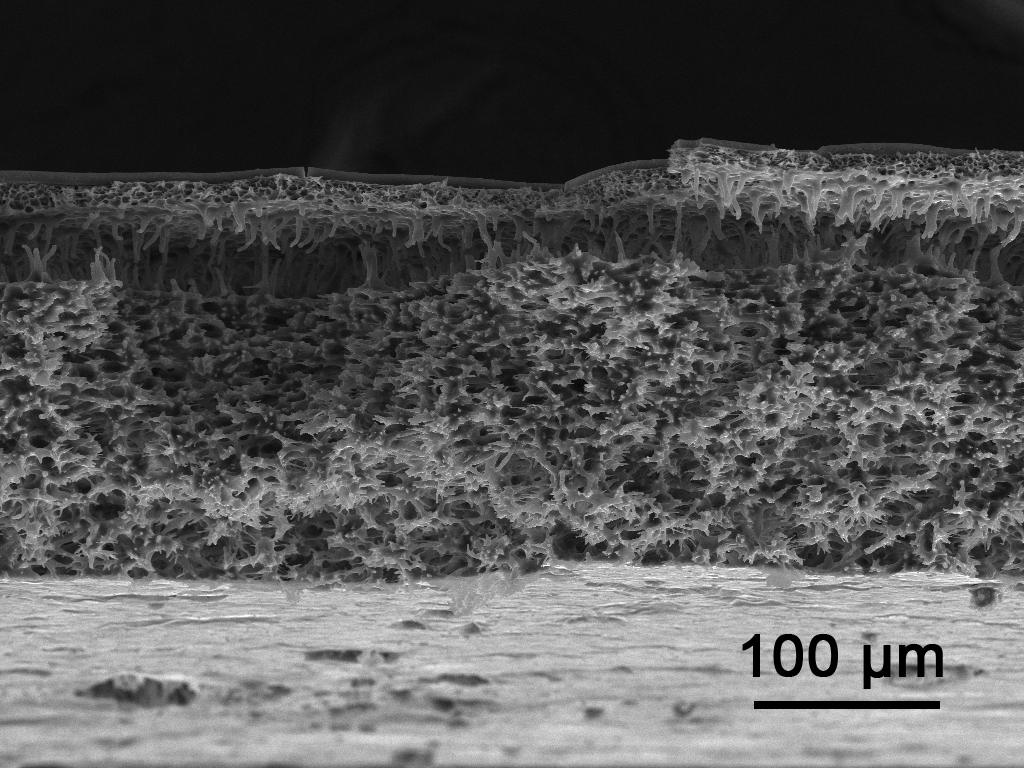


**Supplementary Figure 24.** SEM image of the cross section of the PFSF film with 5 mins solvent exchange time in PNIPAM solution.

When the solvent exchange time is 15 mins, and the cross-section morphology of the film is similar to that of the solvent exchange time of 10 mins, so in order to increase the efficiency of the preparation of the material, the solvent exchange time is determined to be 10 mins (Supplementary Figure 27).


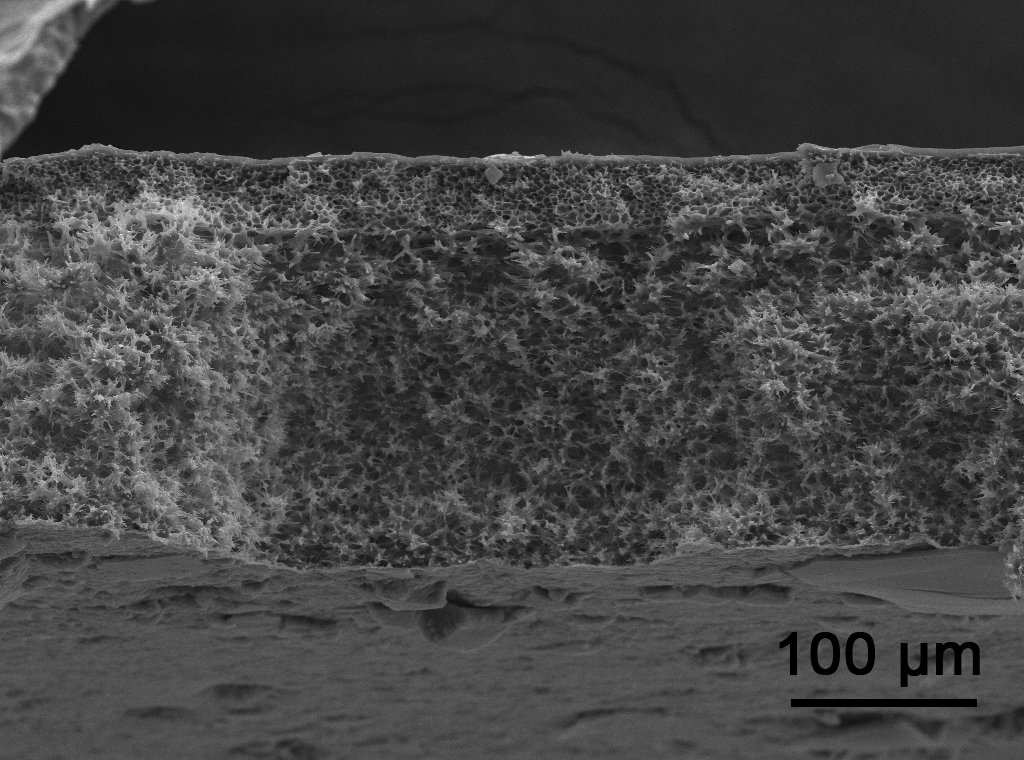


**Supplementary Figure 25.** SEM image of the cross section of the PFSF film with 15 mins solvent exchange time in PNIPAM solution.

**Supplementary Table1.** The fire retardant test results.

| Number of Sample | 1 | 2 | | 3 | 4 | 5 |
| --- | --- | --- | --- | --- | --- | --- |
| Residual flame time after first ignition | 3 | 5 | 6 | | 1 | 2 |
| Residual flame time after second ignition | 0 | 0 | 0 | | 0 | 0 |
| Afterglow time | 0 | 0 | 0 | | 0 | 0 |
| Time to add the afterglow of the experiment after the second flame application | 0 | 0 | 0 | | 0 | 0 |
| The flame or afterglow is not spread to the fixture | No | No | No | | No | No |
| Whether flame particles or droplets ignite cotton pads | No | No | No | | No | No |

**Supplementary Table2.** Thermal properties of the PFSF film used in Energyplus simulation.

| Property | Dry PFSF film | Moist PFSF film | Base mode |
| --- | --- | --- | --- |
| Thickness (mm) | 0.05 | 0.05 | 50 |
| Solar absorptance | 0.1 | 0.61 | 0.8 |
| Visible absorptance | 0.05 | 0.57 | 0.8 |
| Thermal conductivity  (W/mK) | 0.25 | 0.2 | 0.9 |
| Density (Kg/m^3^) | 657 | 1066 | 1121 |

Many existing systems for reflectance modulation require energy inputs, such as electricity, mechanical energy, or liquid circulation systems. This not only increases the overall complexity of the system but also reduces its versatility. Similarly, commonly used PNIPAM hydrogel smart windows require robust encapsulation to protect the fragile gel and prevent water evaporation. In contrast, the PFSF film autonomously adjusts its radiative cooling performance based on the environment, achieving effective temperature regulation without the need for external energy inputs or complex equipment.

**Supplementary Table3.** Summary of various dynamic radiative cooling materials discussed above, their stimuli factors, category, and application.

| Stimuli | | Category | | Material | | Optical properties | | Performance | | | | Application | Ref | |
| --- | --- | --- | --- | --- | --- | --- | --- | --- | --- | --- | --- | --- | --- | --- |
|  |  |  |  |  |  |  |  | Cooling | Heating |  | | |  |  |
| Voltage | | Electro  chromic | | o-WO _3-_*_x_* | | ΔT_lum_=89.1% | | 15.6 | / | Smart windom | | | ^[1]^ | |
|  |  |  |  | Ag^+^/PET-ITO | | ΔT_lum_=72.7%,ΔR_sol_=73.4% | | 1.6 | 17.1 | Building external wall | | | ^[2]^ | |
|  |  | Liquid crystal | | Commercial liquid crystal E7 | | ΔT_sol_=56.5% | | 4.2 | / | Smart windom | | | ^[3]^ | |
| Mechanical force | | Janus Structure | | Acetyl cellulose/ethyl cellulose/carbon black | | R_sol_=96.3%,  E_LWIR_=95.4% | | 8.5 | 2.5 | Building external wall | | | ^[4]^ | |
|  |  |  |  | Vertical graphene/polyacrylonitrile nanofiber | | R_sol_=98.4%, E_LWIR_=92% | | 16 | 9 | Building external wall | | | ^[5]^ | |
|  |  |  |  | MXene-nanofibrils aerogel | | ΔA_sol_=85.5% | | 7 | 4 | Building external wall | | | ^[6]^ | |
|  |  |  |  | PES-Al_2_O_3_/Ti_3_C_2_T_x_ | | R_sol_=97%, E_LWIR_=91% | | 10 | 20 | Textile | | | ^[7]^ | |
|  |  | Mechanical stretch | | TPU nanofibrous membranes | | ΔR_sol_=34.5% | | 10 | 9.5 | Building external wall | | | ^[8]^ | |
| Solvate | | Solvato  chromic | | Porous P(VDF-HFP) | | ΔT_sol_=74% | | 3.2 | 21.4 | Smart window | | | ^[9]^ | |
|  |  |  |  | Delignigied wood | | R_sol_=94.9%, T_sol_=68.4% | | 4.5 | 5.6 | Smart window | | | ^[10]^ | |
|  |  |  |  | Silica microparticles/secreted cellulose nanofiber | | ΔT_sol_=65.3%, E_LWIR_=93.4% | | 3.7 | / | Smart window | | | ^[11]^ | |
| Tempera  ture | | Thermo  chromic Hydrogel | | Poly(N-isopropyacrylamide) | | ΔR_vis_ = 70.0%, ΔT_vis_ = 86.3% | | 3.7 | 5.8 | Smart window | | | ^[12]^ | |
|  |  |  |  | Poly(N-isopropyacrylamide)/Sodium dodecyl sulfate/potassoum tartrate | | ΔT_sol，UCST_ =76.34%, ΔT_sol，LCST_ = 76.75% | | 23.2 | / | Smart window | | | ^[13]^ | |
|  |  | Thermo  chromic Microgel | | (2-anilino-6-(dibutylamino)-3-methylfluoran),/ (bisphenol A) /methyl stearate | | R_sol_=96.8%, E_LWIR_=95.8% | | 3 | 2 | Building external wall | | | ^[14]^ | |
|  |  | Ionic Liquid Gel | | [dhimi][TFSI]/ P(VDF-HFP) | | ΔT_sol_=79.9% | | 5.9 | 0.3 | Smart window | | | ^[15]^ | |
|  |  |  |  | [Bdmim][TFSI]/ [Bmim][TFSI]/SPU | | ΔT_sol_=83% | | 8.8 | / | Smart window | | | ^[16]^ | |
| Tempera  ture | | Moist absorb | | FPAE/PNIPAM | | ΔR=52% | | 10 | 22.5 | Building external wall | | | This work | |

**Reference**

[1] M. Chen, J. Deng, H. Zhang, X. Zhang, D. Yan, G. Yao, L. Hu, S. Sun, J. Zhao, Y. Li, *Adv. Funct. Mater.* **2024**, *30*, 13659

[2] X. Zhao, M. Sheng, H. Tang, H. Pan, C. Guo, D. Zhao, *ACS Appl. Mater. Interfaces* **2024**, *16* (32), 42481.

[3] Y. Deng, Y. Yang, Y. Xiao, H. L. Xie, R. Lan, L. Zhang, H. Yang, *Adv. Funct. Mater.* **2023**, *33* (35), 01319

[4] Z. W. Zeng, B. Tang, F. R. Zeng, H. Chen, S. Q. Chen, B. W. Liu, Y. Z. Wang, H. B. Zhao, *Adv. Funct. Mater.* **2024**, *26*, 03061

[5] H. Yuan, R. Liu, S. Cheng, W. Li, M. Ma, K. Huang, J. Li, Y. Cheng, K. Wang, Y. Yang, F. Liang, C. Tu, X. Wang, Y. Qi, Z. Liu, *Adv. Mater.* **2023**, *35* (18), 09897

[6] W. Yang, P. Xiao, S. Li, F. Deng, F. Ni, C. Zhang, J. Gu, J. Yang, S. W. Kuo, F. Geng, T. Chen, *Small* **2023**, *19* (30), 02509

[7] K. Li, M. Li, C. Lin, G. Liu, Y. Li, B. Huang, *Small* **2023**, *19* (19), 06149

[8] X. Li, Z. Ding, G. E. Lio, J. Zhao, H. Xu, L. Pattelli, L. Pan, Y. Li, *Chem. Eng. J.* **2023**, *461*, 142095.

[9] J. Mandal, M. Jia, A. Overvig, Y. Fu, E. Che, N. Yu, Y. Yang, *Joule* **2019**, *3* (12), 3088.

[10] H. Gao, Y. Li, Y. Xie, D. Liang, J. Li, Y. Wang, Z. Xiao, H. Wang, W. Gan, L. Pattelli, H. Xu, *Composites Part B: Engineering* **2024**, *275*, 111287.

[11] S. Shi, P. Lv, C. Valenzuela, B. Li, Y. Liu, L. Wang, W. Feng, *Small* **2023**, *19* (39), 01957

[12] X. Mei, T. Wang, M. Chen, L. Wu, *Journal of Materials Chemistry A* **2022**, *10* (20), 11092.

[13] Z. Yu, Y. Ma, L. Mao, Y. Lian, Y. Xiao, Z. Chen, Y. Zhang, *Mater. Horizons* **2023**, *20*, 01376.

[14] B. Xiang, P. Xu, R. Li, R. Zhang, *ACS Sustain. Chem. Eng.* **2024**, *12* (2), 841.

[15] Y. Liu, Y. Zhang, T. Chen, Z. Jin, W. Feng, M. Li, L. Chen, C. Wang, *Adv. Funct. Mater.* **2023**, *20*, 07240

[16] B. Li, F. Xu, T. Guan, Y. Li, J. Sun, *Adv. Mater.* **2023**, *35* (20), 11456.
